# Alterations in Lysosome Homeostasis in Lipid-Related Disorders: Impact on Metabolic Tissues and Immune Cells

**DOI:** 10.3389/fcell.2021.790568

**Published:** 2021-12-10

**Authors:** Fernanda Cabrera-Reyes, Claudia Parra-Ruiz, María Isabel Yuseff, Silvana Zanlungo

**Affiliations:** ^1^ Department of Cellular and Molecular Biology, Faculty of Sciences, Pontificia Universidad Católica de Chile, Santiago, Chile; ^2^ Department of Gastroenterology, Faculty of Medicine, Pontificia Universidad Católica de Chile, Santiago, Chile

**Keywords:** obesity, niemann-pick type C (NPC), gaucher disease (GD), lysosomal dysfunction, cathepsins, CD36, B cell activation and membrane contact sites (MCSs)

## Abstract

Lipid-related disorders, which primarily affect metabolic tissues, including adipose tissue and the liver are associated with alterations in lysosome homeostasis. Obesity is one of the more prevalent diseases, which results in energy imbalance within metabolic tissues and lysosome dysfunction. Less frequent diseases include Niemann-Pick type C (NPC) and Gaucher diseases, both of which are known as Lysosomal Storage Diseases (LSDs), where lysosomal dysfunction within metabolic tissues remains to be fully characterized. Adipocytes and hepatocytes share common pathways involved in the lysosome-autophagic axis, which are regulated by the function of cathepsins and CD36, an immuno-metabolic receptor and display alterations in lipid diseases, and thereby impacting metabolic functions. In addition to intrinsic defects observed in metabolic tissues, cells of the immune system, such as B cells can infiltrate adipose and liver tissues, during metabolic imbalance favoring inflammation. Moreover, B cells rely on lysosomes to promote the processing and presentation of extracellular antigens and thus could also present lysosome dysfunction, consequently affecting such functions. On the other hand, growing evidence suggests that cells accumulating lipids display defective inter-organelle membrane contact sites (MCSs) established by lysosomes and other compartments, which contribute to metabolic dysfunctions at the cellular level. Overall, in this review we will discuss recent findings addressing common mechanisms that are involved in lysosome dysregulation in adipocytes and hepatocytes during obesity, NPC, and Gaucher diseases. We will discuss whether these mechanisms may modulate the function of B cells and how inter-organelle contacts, emerging as relevant cellular mechanisms in the control of lipid homeostasis, have an impact on these diseases.

## Introduction

Dyslipidemias are diseases that exhibit an imbalance or abnormal concentrations of lipids such as cholesterol, low-density lipoprotein (LDL) cholesterol (LDL-c), high-density lipoprotein (HDL), cholesterol (HDL-c) and triglycerides. Causes of dyslipidemias are classified as primary and secondary. The first ones are due to mutations in genes coding for proteins related to lipid metabolism and transport. Among the most common primary diseases is familial hypercholesterolemia, which is caused by autosomal dominant mutations in LDL receptors, increasing LDL-c levels (Helkin et al., 2016). Less frequent diseases related to the accumulation of lipids, include the Lysosomal Storage Diseases (LSDs), Niemann-Pick type C (NPC) and Gaucher diseases (Marques and Saftig 2019).

Secondary dyslipidemias are associated with an unhealthy lifestyle, including excessive drug and alcohol consumption, where the most frequent manifestation is obesity, which results from consumption of an unbalanced diet with high fat content (Klop, Elte, and Cabezas 2013). Obesity is a complex chronic disorder with a multifactorial etiology, considered to be an inflammatory disease that results from an excessive accumulation of fat and the disruption of metabolic homeostasis (Lee et al., 2013; Liu and Nikolajczyk 2019). The prevalence of obesity has increased exponentially in all countries in the past decades and has thus become a major heath burden (Popkin and Doak 2009; Blüher 2019).

Obesity-related pathogenesis results in energy imbalance within metabolic tissues, mainly affecting white adipose tissue (WAT) and the liver (Vázquez-Vela, Torres, and Tovar 2008). These tissues also acquire an inflammatory phenotype, where innate immune cells such as macrophages promote inflammation upon exposure to metabolic stress (Cousin et al., 2001; Weisberg 2003; Russo and Lumeng 2018). Inflammation is also promoted by cells of the adaptive immune system, such as B lymphocytes, which manage to infiltrate adipose and liver tissues (Wu et al., 2019), and produce pro-inflammatory cytokines and autoreactive antibodies (Aschermann et al., 2009; Winer et al., 2011; Kao et al., 2015; Srikakulapu and McNamara 2020). Interestingly, inflammation in response to obesity may be B cell-driven, where these cells have been proposed as potential therapeutic targets to overcome this disease (Shaikh et al., 2015).

Cells belonging to metabolic tissues, including adipocytes and hepatocytes, share common pathways that regulate metabolic functions. However, the organelles or cellular pathways within these cells that respond to and are affected by an excess of nutrients, remain incompletely understood. Metabolic functions, and cell signaling are regulated by interactions between the endoplasmic reticulum (ER) and a variety of organelles as well as lipidic structures, including mitochondria, Golgi, lysosomes, the plasma membrane, lipid droplets and the nucleus. There is a growing amount of evidence indicating that obesity leads to dysfunctional interactions between various organelles of different cell types. A prominent example is the dysregulation of mitochondrial dynamics, which affects their associations with the ER, promoting oxidative stress and a imbalance in lipid and glucose metabolism (Bournat and Brown 2010; Arruda et al., 2014; Ejarque et al., 2019).

Additionally, emerging evidence regarding lysosome function, an essential organelle involved in cellular homeostasis suggests that this organelle is susceptible to changes in lipid homeostasis in obesity and LSDs, especially those that accumulate lipids, such as NPC and Gaucher diseases (Dugail 2014; Cermak et al., 2016; Jaishy and Dale Abel 2016; Marques and Saftig 2019). Such dysfunctions can trigger an inflammatory response in adipocytes and hepatocytes, promoting the activation of immune cells and the persistence of a local inflammatory environment (Jaishy and Dale Abel 2016; Ballabio and Bonifacino 2020). Interestingly, B cells in obesity, NPC and Gaucher diseases could also present lysosome dysfunction due to an excess of nutrients, which can lead to alterations in their immune effector functions, such as the degradation and presentation of antigens, which depend on lysosomal activity. However, such functional aspects remain to be investigated.

This review will focus on lysosome homeostasis alterations in lipid-related disorders, particularly in prevalent diseases such as obesity and less frequent NPC and Gaucher diseases. We will discuss the mechanisms involved in lysosomal alterations that are common among cells of metabolic tissues, including adipose tissue and the liver, which are primarily affected in these pathologies. In this context, we will explore common pathways that are altered in the lysosome-autophagic axis, including cathepsins and CD36. We also speculate whether these mechanisms are also perturbed in cells of the adaptive immune system, specifically in B cells, since they rely on lysosomes to promote the processing and presentation of extracellular antigens. Finally, we will address the impact of lysosomal dysfunction on the functionality of MCS in obesity and NPC and Gaucher diseases.

## Alterations in Lipid Metabolism in Liver and Adipose Tissue in Lipid-Related Disorders

Adipose tissue is classified into different types according to its function and appearance; among these are WAT and brown adipose tissue (BAT). WAT acts as an energy store by accumulating free fatty acids (FAs), while BAT is responsible for thermogenesis and energy expenditure (Vázquez-Vela et al., 2008). Adipose tissue is an endocrine organ that undergoes remodeling during metabolic diseases. For example, during obesity, adipocytes, which represent most of the WAT undergo hyperplasia and hypertrophy, as well as cellular death due to hypoxia, infiltration of immune cells with pro-inflammatory phenotypes and high levels of surrounding cytokines (Khan et al., 2020). It is widely described that obesity and its comorbidities are associated with an increased risk of nonalcoholic fatty liver disease (NAFLD) (Neuschwander-Tetri and Brent 2005). This occurs mainly because adipocytes diminish their capacity to store fat, causing chronic elevation of FAs, which are transported by blood circulation to the liver. Lipid accumulation in non-adipose tissues, such as muscle, heart and pancreas, including the liver, as well as an excess in the utilization of FAs cause deleterious effect on glucose metabolism, a term known as lipotoxicity (Engin and Basak 2017; Yazıcı and Sezer 2017). In fact, in liver, FAs are stored as triglycerides in and repackaged as very low-density lipoprotein (VLDL), which then are transported to other tissues, producing global dyslipidemia. Furthermore, in the liver, FAs-induced lipotoxicity promotes ER and oxidative stress, as well as the release of cytokines from inflamed adipose tissue. This promotes inflammation and fibrosis, resulting in progression to nonalcoholic steatohepatitis (NASH) (Peverill et al., 2014; Rada et al., 2020). The increased delivery of FAs to the liver, circulating proinflammatory cytokines, such as TNF-α and interleukin 1β and other bioactive substances, including adipokines and hepatokines, as well as infiltrated immune cells contribute to the appearance of insulin resistance (Kahn and Flier 2000; Jung and Choi 2014; Shi et al., 2019). Overall, obesity causes significant metabolic defects within organs, which have been extensively discussed in previous reviews (Uranga and Keller 2019; Chait and den Hartigh 2020). Altogether, there is a close link between functional changes in WAT that directly affect the liver and vice versa.

Interestingly, changes in lipid metabolism may also contribute to lysosome dysfunction in the liver. Recent studies have underscored the importance of BMP [bis (monoacylglycero) phosphate or lysobisphosphatidic acid, LBPA], a key lysosomal phospholipid in the cellular pathophysiology of patients with lysosomal lipid accumulation, such as obesity and LSDs (Showalter et al., 2020). An increase in the circulating levels of BMP has been described in plasma of patients with NAFLD and NASH, as well as in plasma and livers of mice fed with a high-fat diet (HFD) (Grabner et al., 2019; Showalter et al., 2020). BMP is enriched in late endosomes/lysosomes, where its negative charge plays a key role in the formation of intraluminal vesicles, in lipid and cholesterol sorting, docking structures for the activation of lysosomal hydrolytic enzymes, and degradation of lipids and internal lysosomal membranes (Gallala and Konrad 2011; Pribasnig et al., 2015). In fact, BMP negative charges facilitate the adhesion of soluble positively charged hydrolases, allowing the degradation of lipids at the interface of inner lysosomal membranes (Gallala and Konrad 2011). In LSDs, Showalter et al. (2020) proposed that “accumulation of some glycolipid substrates triggers an adaptive mechanism to bolster BMP levels in an effort to promote the degradation of these species.” Nevertheless, it remains to be determined whether altered levels of BMP are a mediator or a marker of pathological states.

On the other hand, the simplest bioactive phospholipid that is critical in the production and remodeling of intracellular lipids is lysophosphatidic acid (LPA). This phospholipid is implicated in the metabolism of adipose and liver tissues and in the pathogenesis and progression of obesity (Kaffe et al., 2019). In obesity or under conditions of increase lipids, there is an impact on organelle homeostasis and function in adipocytes and hepatocytes, with the lysosome one of the most altered, thus negatively influencing their cellular metabolic function, which will be discussed in the following sections.

### Lysosomal Storage Diseases

LSDs, comprise approximately 70 hereditary diseases produced by mutations in genes encoding for lysosomal hydrolases, transporters or membrane proteins, leading most of the times to accumulation in this organelle of partially degraded substrates within this organelle (Platt et al., 2018). Particularly, LSDs with lipid accumulation show pronounced alterations in lipid metabolism and transport (Platt et al., 2018).

NPC disease is characterized by progressive neurodegeneration and visceral damage caused by mutations in either the *Npc1* (95% of the clinical cases) or *Npc2* genes. Both genes encode for lysosomal proteins involved in cholesterol efflux from lysosomes towards other compartments within the cell (Yañez et al., 2020). Therefore, unesterified cholesterol and other lipids with physicochemical affinity for cholesterol, such as glycosphingolipids, including sphingomyelin, sphingosine and BMP are accumulated in endosomes and lysosomes (Davidson et al., 2009;Neßlauer et al., 2019). Alterations in lysosomal cholesterol transport fail to maintain cellular, tissue, and whole-body lipid homeostasis (Beltroy et al., 2005; Kulinski and Vance 2007). In fact, in the liver, *de novo* synthesis of unesterified cholesterol increases to supply adequate amounts of cholesterol for the synthesis of bile acids or the turnover of membrane sterol (Xie, Turley, and Dietschy 2000; Beltroy et al., 2007). In this context, NPC cells, such as hepatocytes and fibroblast show decreased cholesterol esterification (Soccio and Breslow 2004; Maetzel et al., 2014), which is a key factor, because accumulation of unesterified cholesterol is associated with the infiltration of activated macrophages to metabolic tissues, which produce proinflammatory cytokines and other inflammatory factors and thereby play a critical role in parenchymal cell death (Liu et al., 2007; Liu et al., 2009). Importantly, the nervous system is particularly affected in this pathology, where Purkinje neurons are altered early during the onset of these diseases and are especially sensitive to loss of NPC1 function. Some of the neurological symptoms are associated with their death and early cerebellar degeneration. Moreover, dysfunction of non-neuronal cells in the brain, such as microglia and/or astrocytes, contribute to neurodegeneration (Vanier and Millat 2003). This leads to progressive damage such as generalized neurological deficits including ataxia, dystonia, seizures, and dementia that eventually lead to premature death that characterizes NPC disease (Wraith et al., 2009; Pallottini and Pfrieger 2020). Defects in cells of the nervous system are associated with the accumulation of lipids in lysosomes, which perturbs their interactions with other organelles, their functionality, motility, and cellular distribution, also contributing to a failure in autophagy (Oyarzún et al., 2019). How the accumulation of cholesterol caused by the loss of NPC1 leads to lysosomal dysfunction is not fully understood and will be addressed in this review.

Gaucher disease (GD) is one the most prevalent LSDs and is caused by mutations in the *GBA1* gene, which encodes for the (lysosomal) glucosylceramide degrading enzyme *β*-glucocerebrosidase [also named acid-*β*-glucosidase (GCase)]. GCase deficiency leads to lysosomal storage of glucosylceramide and its deacylated product, glucosylsphingosine. GD has been classified into three types: Type I; is characterized by organomegaly, cytopenia and adult onset non-neuropathic or visceral, Type II and III; both of them have an early onset and progressive compromise brain functions (Nagral 2014; Platt et al., 2018). Cytopenia, splenomegaly and hepatomegaly, result from the infiltration of Gaucher cells, particularly phagocytic macrophages, to the bone marrow, spleen, and liver (Stirnemann et al., 2017; Marques and Saftig 2019). Among alterations reported for liver in GD type I patients, are oscillations in body weight (Kałużna et al., 2019), as well as metabolic abnormalities, including peripheral insulin resistance, dyslipidemia including low levels of high-density lipoprotein (Nascimbeni, Dalla Salda, and Carubbi 2018). Interestingly, a study carried out in patients with GD type 1 revealed high prevalence for liver steatosis (Nascimbeni et al., 2020) and alterations in liver including fibrosis, cirrhosis, and carcinoma (Starosta et al., 2020).

Although GD is considered a multisystemic disease due to the wide spectrum of symptoms, the molecular mechanisms underlying adipose and hepatic tissue alterations caused by the accumulation of glycosphingolipids in lysosomes, remain largely unknown. Most studies have addressed changes at the systemic level, where alterations have been reviewed mainly based on case reports, systematic reviews, and clinical trials (Kałużna et al., 2019). So far, several studies have focused on Gaucher cells (lipid-laden macrophages) that infiltrate liver tissue and the spleen and, in general, there is more information on GD type I, the most common and less aggressive type. In this scenario, current evidence obtained from studies in Gaucher disease suggest that lysosomal dysfunction is due to the accumulation of glucosylceramide and other lipids, such as cholesterol, in this organelle (Yañez et al., 2020). Observations made by immunofluorescence staining of dopaminergic neurons of patients with Parkinson’s that carry mutations in the *GBA1* gene reveal that their lysosomes display a larger size (Kim et al., 2021). Additionally, evidence of lysosomal dysfunction has been reported in a Gaucher neuronopathic murine model after observing changes in the expression of lysosomal marker genes, as well as lower lysosomal biogenesis (Brown et al., 2019). In addition to this, an increase in the presence of multilamellar bodies has been observed in lysosomal structures and perinuclear lysosome clustering in fibroblasts of patients with Parkinson’s disease that carry mutations in the *GBA1* gene (García-Sanz et al., 2017). These alterations in lysosomes also affect the autophagic pathway, where autophagic flux blockage has been observed in Gaucher mouse neurons (Farfel-Becker et al., 2014). Thus, considering that lysosomes are one of the most relevant organelles in sensing the homeostatic state of the cell, a better comprehension of the cellular mechanisms involved in the regulation of lysosome function is essential for the development of new therapeutic approaches to treat LSDs.

### Cellular Pathways Involved in Defective Lysosome-Autophagic Axis

Lysosomes are intracellular organelles essential for the degradation and recycling of macromolecules released by endocytosis, phagocytosis, and autophagy (Appelqvist et al., 2013; Jaishy and Dale Abel 2016). These organelles not only participate in the degradation of molecules but are also highly dynamic complex organelles involved in detecting the state of cellular metabolism, controlling changes between anabolism/catabolism, participating in immune functions, plasma membrane repair, as well as cell adhesion and migration (Ballabio and Bonifacino 2020).

Autophagy provides the required molecular building blocks, such as amino acids, glucose, nucleotides, and FAs, which are used by starving cells. Additionally, autophagy regulates lipid metabolism including FAs oxidation, lipolysis, lipogenesis, ketogenesis, and cholesterol efflux (Martinez-Lopez and Singh 2015; Saito et al., 2019). Lipolysis involves the breakdown of triacylglycerols and esters by cytosolic lipases, while autophagy participates in part of this process, modulating lipoprotein trafficking, as well as, supplying and expanding lipid droplets (LDs) (Zhang et al., 2018a). However, lipid stores can also be accessed *via* lipophagy, a specific subset of selective autophagy that targets LDs and catabolizes their components into free FAs and glycerol (Kounakis et al., 2019). At present, abnormalities in lysosomal and autophagic function are associated with the pathogenesis of metabolic disorders, such as obesity and LSDs (Oyarzún et al., 2019; Wang, et al., 2017).

In this context, several studies have demonstrated the role of autophagy and lysosomes in regulating lipid storage within the two main organs that maintain lipid homeostasis: adipose and liver tissues (Christian, Sacco, and Adeli 2013; Lahiri, Hawkins, and Klionsky 2019). In fact, lipid-related disorders are characterized by a defect in the function of lysosomes that coexists both in the liver and adipose tissue, which negatively influences their metabolic function (Christian et al., 2013). In the next section, we will analyze the resulting lysosomal dysfunction and mechanisms involved including the role of cathepsins and the CD36 receptor as well as its relationship with autophagic functions.

## Lysosomal Dysfunction Coexists in Adipocytes and Hepatocytes in Lipid-Related Disorders: Relevance of Cathepsins, Autophagy and CD36 Alterations

### Role of Cathepsins and Autophagy in Obesity

Several studies regarding lysosomal dysfunction in obese WAT and liver focus on cathepsins and autophagy function, because of their association with lipid storage (Ju et al., 2019; Mizunoe et al., 2019). Cathepsins are a group of proteases involved in intralysosomal protein degradation, which cleave different proteins and polypeptides (McGrath 1999; Turk 2001). These proteases have unique reactive-site properties and a tissue-specific expression pattern (Turk et al., 2012). The most abundant cathepsins (CTS) are L (CTSL) and B (CTSB), which are involved promoting autophagy (Kaminskyy and Zhivotovsky 2012). Moreover, they have been implicated in lysosomal dysfunction in obese murine models in adipose and liver, which display different alterations, such as, oxidative stress, which lead to abnormal lysosomal pH (reduced acidification) (Pascua-Maestro et al., 2017). Such alterations attenuate the maturation of CSTL, causing the accumulation of autophagosomes, and the consequent, suppression of autophagic clearance (Inami et al., 2011; Mizunoe et al., 2017, Mizunoe et al., 2019). Moreover, increased CTSL and decreased CTSB expression at the transcriptional level have been recently observed in abdominal subcutaneous adipose tissue of overweight/obese men and women, but further research is required to establish whether such changes impact protein levels and activity (Xu et al., 2020).

In particular, human obese adipose tissues display high expression levels of autophagic genes, but exhibits attenuated adipocyte autophagic flux (Soussi et al., 2016; Mizunoe et al., 2017). A recent study revealed that omental adipose tissue of obese individuals and adipocytes treated with TNFα, a cytokine secreted within the adipose tissue microenvironment in obesity show an upregulation of lysosomal/autophagic genes (Ju et al., 2019). In contrast, this proinflammatory factor promotes autophagic flux and increases basal lipolysis, impairing triglyceride storage, where CTSB was required for the autophagic process (Ju et al., 2019). In fact, *CTSB* and *CTSD* gene expression are upregulated in obese WAT (Mizunoe et al., 2020). Lipolysis is exacerbated during obesity in WAT and induced overexpression of *CTSB* gene expression in adipocytes displays an increased basal lipolysis (Gaidhu et al., 2010; Mizunoe et al., 2020). Therefore, CTSB has been proposed as a therapeutic candidate for obese WAT. Autophagy is essential for the correct function of adipocytes. Accordingly, it has been reported that inhibition of autophagy by ATG5 or ATG7 knockdown or pharmacological inhibition in preadipocytes, impair their differentiation into mature WAT and lipid storage capacity (Zhang et al., 2016). Nevertheless, it remains to be determined whether upregulation of the expression of genes related to autophagy is sufficient to increase autophagic flux. There is currently a discrepancy regarding the effects on autophagy (enhanced or diminished) in adipose tissue of obese individuals, gene-modified obese animals or diet induced obesity models (García-Barrado et al., 2020). This has been discussed in detail by Zhang et al., 2018b; Zhang et al., 2018a).

Additionally, HFD or FAs exposure induces lysosomal membrane permeabilization in adipose tissue, leading to the release of lysosomal proteases, such as CTSB. The increase of cytosolic CTSB affects mitochondria, increasing ROS production and inducing mitochondrial dysfunction (Gornicka et al., 2012). In fact, *CTSB* ^−/−^ mice showed protection against adipocyte cell death (Gornicka et al., 2012). Interestingly, distinct cellular models have shown that cytosolic CTSB and CTSD participate in the degradation of the pro-apoptotic mediator Bid, which result in its activation and translocation to mitochondria. This translocation leads to cytochrome C release from mitochondria followed by caspase activation, triggering apoptotic cell death (Droga-Mazovec et al., 2008; Yadati et al., 2020). Therefore, we suggest that adipocytes could exhibit a similar mechanism, where cathepsins liberated to the cytosol due to lysosomal impairment induce mitochondrial damage and cell death, thus exacerbating lysosome dysfunction and cell damage. Overall, the role of autophagy and the participation of cathepsins in adipocyte function remains unclear.

In hepatocytes, ER stress alkalinizes lysosomal pH, which reduces the activity of CTSL, CTSB and CTSD, causing the accumulation of autophagosomes and suppressing autophagic clearance, which is associated with hepatic inflammation (Koga et al., 2010; Mizunoe et al., 2019). In line with these findings, autophagy-related proteins were also decreased in the liver of obese mice (Yang et al., 2010; Tong et al., 2019). On the other hand, extracellular CTSD function is relevant in the accumulation of hepatic lipids and intracellular CTSD is involved in essential processes, such as mitochondrial oxidative phosphorylation and electron transport function (Yadati et al., 2021). Recently, it was described that administration of the extracellular CTSD inhibitor reduced hepatic triglyceride levels in mice fed with a HFD, whereas intracellular or the extracellular CTSD inhibitor decreased hepatic cholesterol levels (Yadati et al., 2021). With these results the authors concluded that extracellular CTSD is involved in pathways related to lipids and inflammation.

Similar to adipocytes, a HFD also induces lysosomal membrane permeabilization and lipotoxicity in the liver of mice with NASH and NAFLD (Feldstein et al., 2006; Fucho et al., 2014). It has been reported that lysosome permeabilization is mediated by Bax, a pro-apoptotic mediator, which induces the release of cytosolic cathepsins (Feldstein et al., 2006). This results in caspase activation or mitochondrial membrane permeabilization mediated by caspase activation, triggering apoptosis and liver injury (Feldstein et al., 2006; Fucho et al., 2014; Jaishy and Dale Abel 2016).

Overall, these reports suggest that in obesity, autophagy is mostly downregulated in adipose tissue and in the liver. Also, that cathepsins are relevant in mediating the autophagy process and their release to the cytosol contributes to lysosome and cellular dysfunction through mitochondrial damage. However, more studies are needed to clarify the role of cathepsins in lysosomal dysfunction in these tissues in the context of obesity.

At present, several studies have focused on alterations in lysosomal distribution and dynamics, motility, and autophagic function involved in a variety of conditions, such as neurodegenerative diseases, cancer, and obesity (Seranova et al., 2017; Oyarzún et al., 2019). However, as expected, lysosomal dysfunction is also a common feature in LSDs, and their functional implications will be discussed in the following section.

### Role of Cathepsins and Autophagy in Niemann-Pick Type C

This section will focus on the role of cathepsins and autophagy in hepatocytes in the context of NPC and Gaucher diseases, considering that most of the studies have been performed in the liver. An increase in the expression of CTSB, CTSD, CTSS, and CTSZ were recently observed in the liver and hepatocytes of *Npc1* ^*−/−*^ mice (Balboa et al., 2021; van der Lienden et al., 2021), suggesting that lysosomal proteases were increased. However, *Npc1* ^−/−^ mouse embryonic fibroblasts showed increased levels of mature CTSB and CTSD and normal lysosomal proteolytic functions, suggesting that they remained unaffected (Sarkar et al., 2013). Interestingly, impaired clearance of autophagosomes has been observed in human dermal fibroblasts with mutations in *Npc1* and fibroblasts of *Npc1* ^−/−^ mice, which correlated with an inhibition of lysosomal protease activity produced by stored lipids (Elrick et al., 2012). In fact, NPC1-deficient lysosomes derived from HEK293T cells have proteolytic defects, where inhibition of mTORC1 by genetic and pharmacologic manipulation restores lysosomal proteolysis without correcting cholesterol storage (Davis et al., 2021). Regarding autophagic vesicle accumulation, an increase in levels of LC3-II (light chain 3 of microtubule-associated protein 1), a specific autophagosome maker, has been reported in the cerebellum, the hippocampus and livers of *Npc1* ^−/−^ mice as well as mouse embryonic fibroblasts (Pacheco et al., 2007; Sarkar et al., 2013; Meske et al., 2014). In addition, NPC1 iPSC (patient-specific induced pluripotent stem cells) show dysfunctional autophagic flux, where LC3-II and p62, an autophagy adaptor protein responsible for cargo delivery of ubiquitinated substrates, were significantly increased (Maetzel et al., 2014). However, it remains unclear whether an increase in the number of autophagosomes results from an increase in autophagic activity or a reduction in autophagy flux caused by impaired autophagosome-lysosome fusion (Dai et al., 2017). On the other hand, lysosome membrane permeabilization has been observed in NPC disease (Chung et al., 2016). As we have described before, lysosomal permeabilization promotes cytosolic release of CTSD, which triggers apoptosis in adipose and liver tissues of mice fed with a HFD. Interestingly, hippocampal neurons incubated with U18666A (a classic NPC1 inhibitor) have increased levels of *CTSD* mRNA and enzyme activity, which is associated with neuronal apoptosis (Amritraj et al., 2013). However, it has been reported that early lysosomal cholesterol accumulation induced by U18666A in human fibroblast attenuates apoptosis by preventing lysosome permeability and reducing CTSD release from lysosomes (Appelqvist et al., 2011). Cholesterol overload ultimately triggers lysosome membrane permeabilization, which disrupts lysosome homeostasis. Hence, we speculate that NPC livers could exhibit a similar mechanism thus contributing to lysosome dysfunction, but this remains to be investigated.

### Role of Cathepsins and Autophagy in Gaucher Disease

**FIGURE 1:**
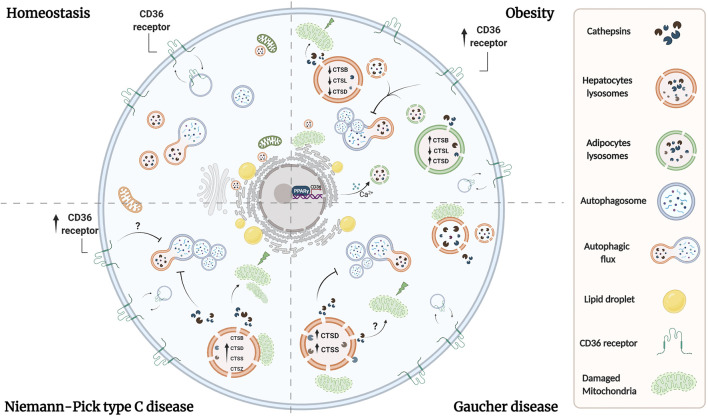
Lysosomal dysfunction coexists in adipocytes and hepatocytes in lipid-related disorders. Overload of lipids induces permeabilization of lysosomal membrane in both adipose and liver tissues, leading to the release of lysosomal proteases. Reports have shown that cytosolic proteases increase ROS production and induce mitochondrial dysfunction, triggering apoptosis and liver injury. Whether similar alterations exist in adipocytes under these conditions, remains to be evaluated. Additionally, a imbalance in cathepsins levels exists in both tissues in obesity. Overall, obesity is associated with oxidative stress, which lead to alkalinization of lysosomal pH, causing the accumulation of autophagosomes, and suppression of autophagic clearance. In NPC and Gaucher diseases there are alterations in the levels or activity of cathepsins which are associated with proteolytic impairment and inhibition of autophagy, which altogether contribute to lysosome dysfunction probably by analogous mechanisms as those observed in obesity. On the other hand, dyslipidemia is associated with an increase in the expression of CD36 in adipocytes and hepatocytes, which leads also to an increase in lysosomal pH and an inhibition of autophagy. In adipocytes, CD36 mediates lysosomal calcium overload through the ER, and we speculate that a similar mechanism could occur in hepatocytes. Additionally, lipid uptake is mediated by an increase in CD36 expression in adipocytes and hepatocytes by PPAR-*γ*, which promotes lipid accumulation and contributes to lysosome dysfunction. Although the upregulation of CD36 in NPC liver has been observed, its role in NPC and Gaucher hepatic diseases remains unclear.

Cathepsins have not been extensively studied in GD. However, in neuronopathic forms of GD changes in the subcellular distribution of CTSD have been detected in the brain and as well as an increase of CTSD in areas with neuronal loss, astrogliosis, and microgliosis, suggesting a role for CTSD in neuronal injury (Vitner et al., 2010). Also, GD mouse models show an increase of CTSD and CTSS in the liver and spleen, whereas patients with GD show increased serum levels of both proteases (Mistry et al., 2010; Afinogenova et al., 2019). Similar to NPC disease, there is evidence suggesting that autophagy is defective in GD. Primary fibroblasts deficient in saposin C have impaired autophagosome degradation associated with reduced CTSB and CTSD activity (Tatti et al., 2012; Seranova et al., 2017). Defects in the maturation and accumulation of autophagosomes including autophagic cargo were found in neurons and astrocytes cultured from mice deficient for glucocerebrosidase, prosaposin or glucosylceramidase (Farfel-Becker et al., 2014; Seranova et al., 2017). Additionally, LAMP2 and p62 accumulate in the brain of neuronopathic GD mouse models suggesting that autophagosome/lysosome function is compromised (Sun et al., 2010). In contrast, fewer autophagic vacuoles have been reported in peripherical blood mononuclear cells derived from GD patients with an increase of cytoplasmic localization of LC3A/B. This was accompanied by lysosome accumulation suggesting that constitutive autophagy is inactivated (Ivanova et al., 2019). In addition, neuronal mouse models of glucocerebrosidase deficiency showed a redistribution of CTSD from the lysosome to the cytosol suggesting that these cells also contain lysosomes with permeabilized membranes (Serrano-Puebla and Boya 2016). Similar to what was discussed in NPC disease, this cytosolic cathepsin may be promoting the mitochondrial damage that is observed in Gaucher disease (Cleeter et al., 2013; Osellame et al., 2013). Altogether, these data indicate common mechanisms coexisting among these diseases, where the functional deterioration of cathepsins is associated with impaired autophagy and their cytosolic distribution by lysosome membrane permeabilization is directly linked with lysosomal dysfunction and cellular damage (Figure 1).

### Lysosomal Dysfunction in Obesity, Role of CD36 in Adipocytes and Hepatocytes

CD36 is a multifunctional immuno-metabolic receptor that belongs to the family of class B scavenger receptors. This receptor is primarily localized in caveolae and mediates FA uptake by endocytosis (Hao et al., 2020). This glycoprotein is widely expressed in tissues and different cell types, including adipocytes, hepatocytes, macrophages, monocytes, platelets, among others (Silverstein and Febbraio 2009). Scavenger receptors recognize modified self-antigens and are defined by their ability to bind oxidized-LDL, which is relevant in atherosclerosis pathogenesis, where the formation of lipid-laden foam cells promotes atherosclerotic plaques (Febbraio and Silverstein 2007; Silverstein and Febbraio 2009; Tian et al., 2020). In particular, CD36 binds these and other oxidized phospholipids, long-chain FA, and thrombospondin and its function varies according to each cell type (Gillotte-Taylor et al., 2001; Silverstein and Febbraio 2009). Evidence indicates that CD36 is not only a FA transporter but also an essential regulator of intracellular FA and immune homeostasis and has emerged as a relevant player connecting lysosomal dysfunction and lipid homeostasis alterations (Pepino et al., 2014; Rawnsley and Diwan 2020; Tian et al., 2020).

CD36 plays an important role in liver lipid homeostasis, lipophagy and autophagy, and its levels increase in hepatocytes exposed to high-fat diets as well as in hepatic steatosis and NAFLD (Bechmann et al., 2010; Love-Gregory and Abumrad 2011; Miquilena-Colina et al., 2011; Li et al., 2019). Indeed, in obesity, lipid accumulation and lysosomal dysfunction in adipocytes and hepatocytes depends on the expression and role of CD36 (Koonen et al., 2007; Li et al., 2019; Rawnsley and Diwan 2020).

A recent study described an increase in the expression of CD36 in preadipocytes of mice fed with a HFD and also in obese patients (Luo et al., 2020). At a cellular level, CD36 was shown to interact with Fyn leading to the phosphorylation and activation of IP3R1 [inositol (1,4,5)-trisphosphate receptor 1], in FA-treated adipocytes. Consequently, an excess of calcium is transported from the ER to the lysosomes, generating an increase in lysosomal pH and in the production of inflammatory cytokines, while decreasing lipophagy and impairing lysosomal function (Luo et al., 2020; Rawnsley and Diwan 2020). Accordingly, lysosomal disruption is promoted by CD36/Fyn/IP3R1-mediated lysosomal calcium overload, which can be associated with a failure in autophagic flux observed in adipocytes of obese mice (Mizunoe et al., 2017). Additionally, activation of PPAR-γ (peroxisome proliferator-activated receptor *γ*), a nuclear receptor responsible for adipocyte differentiation and adipogenesis mediates FAs uptake through an increase of CD36 expression (Tontonoz and Spiegelman 2008; Cai et al., 2012). It has been reported that CD36 contributes to inflammation and cell death in adipose tissue of mice fed with a HFD (Cai et al., 2012). These findings indicate that CD36 participates in mediating the alteration of lysosomal calcium homeostasis and uptake of lipids in adipocytes, which leads to an alteration in autophagy and lysosomal function.

On the other hand, an increase in plasma LPA levels has been reported in mice fed with a HFD, which is associated with an increase in adipose tissue *ATX* (autotoxin) mRNA levels (Dusaulcy et al., 2011). Extracellular LPA is mainly produced from lysophosphatidylcholine by lysophospholipase D activity of ATX. Thus, LPA levels are closely related to the ATX protein content and/or activity (D’Souza et al., 2018; Ferry et al., 2003). The ATX-LPA pathway may contribute to obesity-induced insulin resistance by stimulating fibrosis, inflammation, and/or suppressing BAT, mitochondrial function and impairing PPAR-*γ* expression and activity. This last idea is supported by studies showing that mice with *ATX* deletion fed with an obesogenic diet present an increase in *PPAR-γ* mRNA levels (Dusaulcy et al., 2011; D’Souza et al., 2018). Although, the data suggest that the ATX-LPA axis reduces PPAR-*γ* function, the specific mechanisms by which it contributes to obesity remains to be elucidated (Jose and Kienesberger 2021). In contrast, it has been reported that obese individuals have higher *PPAR-γ* mRNA levels, which contributes to an increase in the numbers of adipocytes (Vidal-Puig et al., 1997; McCann and Ratneswaran 2019). Interestingly, activation of PPAR-*γ* induces an increase in the number of small and insulin-sensitive adipocytes and up-regulates adiponectin, improving insulin sensitivity in the liver and muscle (Jakab et al., 2021). Therefore, more research is needed to address the precise role of ATX-LPA signaling and the PPAR-*γ* function in adipose tissue under obesity.

Additionally, studies have shown that the expression of CD36 under a HFD negatively regulates autophagy in hepatocytes. In mice with NASH, translocation of CD36 to the plasma membrane in hepatocytes is associated with lower AMPK (adenosine monophosphate-activated protein kinase) activity and lower FA oxidation (Zhao et al., 2018). Conversely, CD36 knockout mice fed with a HFD show increased autophagy/lipophagy, which promotes lipolysis and FAs catabolism by β-oxidation to produce energy, attenuating the accumulation of lipids (Li et al., 2019). In this report, the authors suggest that CD36 deficiency results in an increase in autophagy, which is correlated with a rise in the translocation of TFEB to the nucleus. Indeed, an increase in nuclear TFEB was observed upon knockdown of CD36 in human hepatoma cells in the presence of palmitic acid; however it was not quantified. In line with this work, inhibition of the internalization of CD36 by a deficiency of SNX10 (Sorting Nexin 10, a protein involved in protein sorting and membrane trafficking in endosomes) in lipid tissue-resident macrophages, suppresses the Lyn-AKT signaling pathway, which results in increased translocation of TFEB to the nucleus and enhances the function of the autophagy-lysosome system (Fan et al., 2020; You et al., 2020). Overall, one could speculate that, under obesity, translocation of TFEB could be inhibited in hepatocytes, thus decreasing the expression of genes related to lysosomal biogenesis and autophagy. This also suggests that an increase in CD36 expression in hepatocytes could cause an alteration of lysosomal calcium homeostasis as observed in adipocytes, enhancing lysosomal dysfunction.

Interestingly, the levels of CD36 in the liver are much higher in *ATG5* ^−/−^ mice, suggesting that the autophagy machinery also regulates CD36 expression (Li et al., 2018). Alternatively, in adipocytes, silencing of ATG5 led to a deterioration in the accumulation of triglycerides during adipogenesis and the inhibition of autophagy (Singh et al., 2009; Clemente-Postigo et al., 2020). The latter suggests that, in obesity, an increase in the expression of CD36 in adipocytes may be dependent on ATG5, which would contribute to the inhibition of autophagy through the aforementioned mechanisms.

On the other hand, elevated levels of plasma FA, induced by FA-rich diets, contribute to hepatic insulin resistance, increased glucose production and hepatic steatosis (Seppala-Lindroos 2002). Accordingly, it was shown that hepatocytes from obese rats require high insulin levels to translocate CD36 to the plasma membrane to improve the uptake of FA and the synthesis of triglycerides (Buqué et al., 2012). The authors of this work propose that hyperinsulinemia present in animal models and patients with insulin resistance and fatty liver may contribute to an increase in the expression of CD36 and in the accumulation of fat in the liver (Buqué et al., 2012). Conversely, CD36 deficiency decreased insulin resistance in primary adipocytes isolated from HFD-fed mice (Kennedy et al., 2011; Luo et al., 2020). Thus, based on the above, the expression of CD36 would also modulate the levels of insulin resistance. Consequently, high insulin levels observed in obesity may contribute to lysosomal dysfunction by generating an increase in the expression CD36, which together contribute to obesity-associated dyslipidemia.

Alternatively, similar to what was discussed in adipose tissue, several studies have shown that the hepatic expression of CD36 is positively regulated by activation of PPAR-γ under conditions of nutrient overload (Jung, Zhou, and Xie 2008; Pettinelli and Videla, 2011; Wang et al., 2020a; Yu et al., 2021). Interestingly (Yu et al., 2021), showed that hepatic extracellular galectin-3 promotes fatty acid uptake through CD36 in a PPAR-γ pathway-dependent manner (Yu et al., 2021). Indeed, galectin-3 is a lectin involved in liver inflammation, fibrosis, and related metabolic disorders (Iacobini et al., 2011; Pejnovic et al., 2013). Moreover, it has been reported, that hepatic extracellular galectin-3 is upregulated in NASH and its inhibition in mice fed with a HFD, reduced hepatic CD36 expression, the accumulation of lipids and hepatic steatosis (Iacobini et al., 2011; Yu et al., 2021). These findings indicate that CD36 expression in obese liver tissues is regulated by activation of PPAR-γ through galectin-3.

On the other hand, it has been reported that LPA is an agonist of PPAR-*γ* (McIntyre et al., 2003). LPA upregulates CD36 expression on the surface of monocytes through PPAR-*γ* stimulation and induces lipid accumulation through oxidized-LDL absorption (McIntyre et al., 2003). However, this mechanism has not yet been described in hepatocytes. Interestingly, LPA is involved in the progression of liver fibrosis, so it has been proposed as a therapeutic target (Kaffe et al., 2019). Therefore, we suggest that LPA (which increases with the overload of lipids) could participate as an agonist of PPAR-*γ*, promoting an increase in fatty acid uptake by CD36 in hepatocytes. Nevertheless, more research is required to demonstrate whether this mechanism contributes to lysosome dysfunction in obesity.

Concerning NPC disease, proteomic analysis from hepatocytes of *Npc1* ^−/−^ mice, performed by our group, showed an increase in the levels of CD36 protein levels (Balboa et al., 2021). Increased transcript levels of the CD36 in hepatocytes of *Npc1* ^−/−^ mice have been observed by our group and others (Vázquez et al., 2011; Dos Reis et al., 2020). Intriguingly, galectin-3 is increased in liver tissues from *Npc1* ^−/−^ mice (Cluzeau et al., 2012). In this sense, we propose that this galectin could be mediating lysosomal dysfunction by increasing CD36 expression through the PPAR-*γ* pathway.

Alternatively, LPA accumulation in liver tissues of *Npc1* ^−/−^ zebrafish, which reproduces the pathological features of NPC disease has been reported (Lin et al., 2018). Recently, lipidomic studies of liver tissue from *Npc1* ^−/−^ mice showed an increase of BMP (Pergande et al., 2019). However, they do not analyze the levels of LPA. Based on this, it is possible to speculate that the hepatic levels of LPA increase in *Npc1* ^−/−^ mice. Under this scenario, we suggest that LPA may also promote the expression of CD36 in NPC hepatocytes through the PPAR-*γ* pathway (similar to what was discussed in obesity), promoting lysosome dysfunction. However, this requires further investigation.

On the other hand, BMP levels are increased in fibroblasts pretreated with U18666A, in fibroblasts derived from NPC1 patients, and fibroblasts and livers of *Npc1* ^−/−^ mice (Appelqvist et al., 2011; Moreau et al., 2019; Ilnytska et al., 2021). Interestingly, NPC1-deficient human fibroblasts incubated with BMP show a reduction in lysosomal cholesterol levels, which was associated with a direct interaction between BMP and NPC2, leading to an increase in lysosomal cholesterol efflux (McCauliff et al., 2019). This suggests that the increase in BMP compensates lipid accumulation at early stages until BMP production and the endosomal system collapse under lipid overload (Liu et al., 2014).

Interestingly, BMP levels were shown to be elevated in skin fibroblasts and plasma samples from patients with Gaucher disease (Meikle et al., 2008). However, its relevance remains unclear. Additionally, there are few studies that have evaluated the levels of LPA in Gaucher disease. LPA plasma levels seem to increase in Gaucher patients, but the results are not conclusive due to the low number of samples (Byeon et al., 2015). Overall, alterations in the expression or function of CD36 have not been reported in adipocytes in NPC or in Gaucher disease. Nevertheless, based on the evidence described in obesity, we speculate that CD36 expression and function could also be compromised in GD adipocytes and hepatocytes. Therefore, elucidating the role of CD36 may contribute to a better understanding of the dysregulated lysosomal function observed in both diseases.

Taken together, these findings show that in obesity, adipocytes and hepatocytes express higher levels of CD36, which leads to defective lysosome homeostasis and negatively regulates autophagic function. Thus, it is possible to speculate that CD36 could participate in the modulation of lysosomal dysfunction in NPC and Gaucher diseases (Figure 1). However, the mechanism by which these processes are controlled, requires further investigation.

## Role of Lysosomes in the Immune Response of B Cells: Impact of Lipid-Related Disorders

In recent years, several studies have suggested that B cells are also involved in adipose and liver tissues inflammation contributing to the pathogenesis of obesity. B cells are activated in adipose tissue during obesity (Shaikh et al., 2015; Srikakulapu and McNamara 2020) and in HFD-fed mice, these cells migrate to the liver, promoting inflammation, where macrophage differentiation to pro-inflammatory phenotypes secrete pro-inflammatory cytokines (Wu et al., 2019). Additionally, intrahepatic B cells might be involved in NAFLD by secretion of pro-inflammatory cytokines and IgG2a, a potent inducer of antibody-based inflammation (Zhang et al., 2016). Importantly, lysosomal function is critical for B cell activation during antigen recognition (Obino et al., 2017; Sáez et al., 2019). The question then arises as to whether such B cell functions are affected in obese patients. It is then necessary to understand how B cell activation occurs and the importance of lysosomes during this process.

**FIGURE 2:**
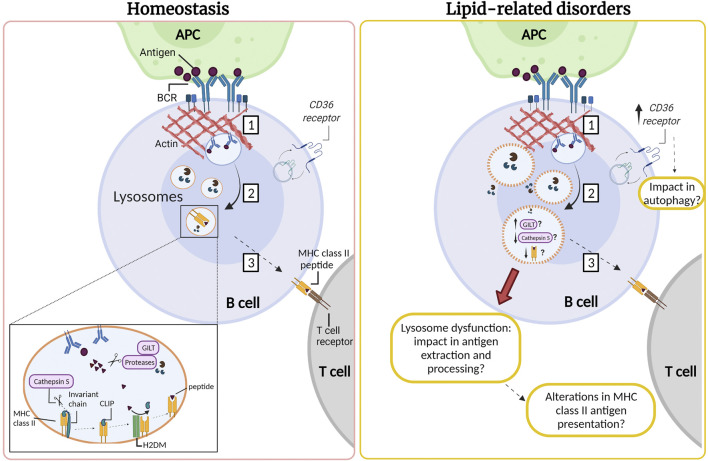
Lysosomes are required for antigen processing in B cells: impact of lipid-related disorders. (1) The interaction of the B cell receptor (BCR) with antigens tethered at the surface of an antigen presenting cell (APC) gives rise to an immune synapse. Activation of the BCR triggers signaling cascades which induce extensive remodeling of the actin cytoskeleton at the synaptic interface, promoting membrane extensions and efficient BCR-antigen internalization into late endosomal compartments. (2) BCR-antigen converges into lysosomes which contain the accessory molecules, such as GILT, H2DM, proteases and MHC class II. MHC class II molecules are associated with the invariant chain, which undergoes proteolysis by cathepsin S generating a smaller fragment called CLIP. Subsequently, the H2DM chaperone catalyzes the exit of CLIP and the loading of antigenic peptides into the MHC-II pocket. (3) Next, MHC-II molecules are transported to the surface of the B cell to be presented to the CD4^+^ T lymphocyte to promote B-T cell cooperation. We speculate that B cells that infiltrate the inflamed adipose and liver tissue in lipid-related disorders could also present lysosomal dysfunction resulting in lower cathepsin S levels or activity. Moreover, B cells might exhibit permeabilization of their lysosome membrane, similarly to observations in other cell types in lipid-related disorders. We suggest that in obesity there could also be an increase in the expression of CD36, which may be enhanced upon B cell activation. This might impact in autophagy, enhancing the canonical pathway and diminishing noncanonical autophagy. Such defects could impact the capacity of B cells to extract and process antigens, which relies on lysosome integrity. However, these functions remain to be evaluated and the question that arises is how are lysosomes in B cells affected by an excess of nutrients in obesity and LSDs?

B cell activation occurs when the B cell receptor (BCR) recognizes immobilized antigens on antigen-presenting cells, triggering an immune synapse. Activation of the BCR induces a signaling cascade that promotes the recruitment of lysosomes to the synapse, which depends on centrosome repositioning. These lysosomes fuse with the synaptic membrane, secreting their acidic content, facilitating the extraction, and processing of antigens. Activation of B cells induces an increase in the synthesis of MHC-II (type II major histocompatibility complexes) (Lankar et al., 2002; Yuseff et al., 2011). The synthesis of these molecules begins in the ER, where the αβ dimers of MHC-II are associated with an invariant chain that prevents binding to peptides and promotes their transport towards endo-lysosomes (Roche and Cresswell 1990). In this compartment, the invariant chain undergoes proteolysis by CTSS, an asparaginyl endopeptidase, generating a smaller fragment called CLIP. Subsequently, the H2DM chaperone catalyzes the exit of CLIP and the loading of the generated peptides into the MHC-II pocket (Lankar et al., 2002; Blum, Wearsch, and Cresswell 2013). Once the peptides are assembled, the MHC-II molecules are transported to the surface of B cells to be presented to the CD4^+^ T lymphocytes to promote B-T cell cooperation (Lanzavecchia 1985; Mitchison 2004). This allows co-stimulation and proliferation of both cells and the differentiation of B cells into plasma cells that produce specific antibodies (Harwood and Batista 2010; Yuseff et al., 2011). The impact of lipid accumulation in lysosome function, as well as, in antigen extraction and presentation by B cells, remains to be addressed (Figure 2).

### Role of CD36 in B Cells in Obesity

As previously mentioned, CD36 is expressed in adipocytes and hepatocytes, but has also been detected in immune cells, such as macrophages and dendritic cells as well as T and B cells (Urban et al., 2001; Corcoran et al., 2002; Won et al., 2008; Couturier et al., 2019). A study published recently by He et al. (2021) demonstrated that all peripheral human blood B cell populations express intracellular CD36 except naïve B cells (He et al., 2021). They found that CD36 colocalizes with LC3B upon the induction of autophagy and splenic B cells increase CD36 expression and autophagosome formation after LPS stimulation *in vitro*. Interestingly, B cells from CD36^−/−^ mice have less autophagosome formation upon LPS stimulation (He et al., 2021) and exhibit defects in mitochondrial mobilization and also oxidative phosphorylation (He et al., 2021), as well as, reduced plasma cell formation, subsequent antibody production and proliferation. Remarkably, the expression of CD36 increases in T lymphocytes of adipose tissue and in the liver of mice fed with a HFD, but it is unclear whether it is preferentially expressed by a specific subset of cells (Couturier et al., 2019). However, the authors propose that there could also be an increase in the expression of CD36 in natural killer and B cells in these tissues (Couturier et al., 2019).

As mentioned above, CD36 increases its expression under a HFD inhibiting autophagy in adipocytes and hepatocytes. In obesity, we speculate that B cells could also display elevated expression levels of CD36, which could regulate autophagy during their activation. B cell activation triggers a temporary change from basal to non-canonical autophagy, which is essential to control B cell differentiation (Martinez-Martin et al., 2017). Under these conditions, components from the autophagic machinery can be recruited to other pre-existing membranes, different from the phagophore, where they generally reside (Martinez-Martin et al., 2017). In this sense it has been described that ATG5 is necessary for the internalization and trafficking of BCR towards LAMP1 and MHC-II positive compartments, as well as for the optimal presentation of antigens to T cells (Arbogast et al., 2019). Additionally, it has been shown that activation of B cells with BCR ligands produces the colocalization of LC3 with the BCR and with MHC-II vesicles, showing an association of autophagic vesicles involving BCR and the MHC-II-mediated antigen presentation (Ireland and Unanue 2011). Therefore, it is tempting to speculate that in obesity there is an increase in the expression of CD36, which may be enhanced upon B cell activation. This may cause an imbalance in autophagy, enhancing the canonical pathway and diminishing noncanonical autophagy. In this sense, this could alter the lysosomal function, impairing the processing and presentation of antigens to T cells, as well as plasma cell formation and subsequent antibody production.

In contrast, B cells are also involved in adipose and liver tissue inflammation contributing to the pathogenesis caused by obesity. Several studies have shown that B cells in adipose and liver tissues in HFD-fed mice, enhance the activation of CD4^+^ T lymphocytes and their differentiation into T helper (Th) 1 cells (Winer et al., 2011; Zhang et al., 2016). Interestingly, it has been reported that obese patients have low levels of PPAR-*γ* mRNA in peripheral blood mononuclear cells and protein levels in serum (Ramon et al., 2012). PPAR-*γ*-deficient T cells are hyperreactive to T cell receptor stimulation, which promotes greater B cell activation, thereby leading to autoantibody production (Park et al., 2014). We speculate that an imbalance in PPAR-*γ* activity in B cells in obesity could lead to the aforementioned defects. Thus, clarifying the role of PPAR-*γ* and its functional relationship with CD36, should help elucidate how a lipid overload impacts B cell activation, and affects antigen presentation to CD4^+^ T lymphocytes.

### Role of Cathepsins in B Cells in Obesity

Based on the role of cathepsins involved in antigen processing, it has been reported that gamma-interferon-inducible lysosomal thiol reductase (GILT) facilitates antigen processing since it reduces the disulfide bonds of proteins in the endo-lysosomal compartment. It has been hypothesized that the reduction of protein disulfide bonds that pass through the endocytic pathway may facilitate the processing of hidden epitopes so that they are not restricted by MHC-II (Singh and Cresswell 2010). As described previously, CTSS is essential for MHC-II processing and has disulfide bonds susceptible to this reduction since it is found in the lysosome together with GILT (Phipps-Yonas et al., 2013). Expression of GILT in primary B cells derived from mice decreases the expression and activity of CTSS but does not substantially alter the expression of other lysosomal proteins, such as H2DM, H2DO and CTSL (Phipps-Yonas et al., 2013). Interestingly, a transcriptomic study showed that the gene encoding for GILT was 1.72 times more expressed in the omental adipose tissue of severely obese men with metabolic syndrome compared to those without the syndrome (Turcot et al., 2012). Therefore, it is possible to speculate that dyslipidemia caused by obesity could induce an increase in the expression of GILT, and consequently a defect in lysosomal function by reducing the expression and activity of CTSS. Consistent with the previous idea, it has been reported that antigen presentation is defective in B cells derived from *CTSS* ^−/−^ or *CTSL* ^−/−^ Mice (Nakagawa et al., 1999). Additionally, CTSS regulates the level of mature CTSL in B cells, since it was shown that CTSL levels increase in the absence of CTSS, but in this study the activity of this enzyme was not detected (Honey et al., 2001). Thus, it is possible that a lower expression of CTSS could induce a dysregulation of CTSL, which could lead to a decrease in antigen processing, also altering the presentation of antigenic peptides on MHC-II to the T cells. As described in the previous section, reduction in levels of CTSL has been observed in adipose tissue and the liver in obesity. On the other hand, similar to what was discussed in obesity, B cells might exhibit permeabilization of their lysosome membrane. Such defects could impact the capacity of B cells to extract and process antigens, which relies on lysosome integrity. Based on these studies, it would be relevant to study the role of CD36 and/or cathepsins in B cell function associated with obesity.

### Alteration of B Cell Functions in NPC and GD Diseases

In the majority of LSDs the pathology is primarily neuronal, but the immune system has also been implicated and predisposed towards suppression (Castaneda et al., 2008; Platt et al., 2016; Rigante et al., 2017). Lysosomal glycosphingolipid storage increased has been shown in splenic B cells derived from *Npc1* ^*−/−*^ mice and peripheral B cells from NPC1 patients (Lachmann et al., 2004; Vruchte et al., 2010). Additionally, results from our group showed that B lymphocytes treated with U18666A, exhibit lysosomal accumulation of unesterified cholesterol (Oyarzún et al., 2019) The lysosomes of NPC cells show a typical and concentrated perinuclear pattern, which results from an increase in the reverse transport of lysosomes, and their perinuclear clustering (Oyarzún et al., 2019). This is a key factor, because the correct distribution and motility of lysosomes promote a functional immune synapse between B cells with antigen-presenting cells. Also, the fusion of endolysosome compartments required to facilitate antigen uptake from presenting cells, is critical to achieve an efficient adaptive immune response (Yuseff et al., 2015). In fact, an excess of lipids in lysosomes of B cells might also promote the permeabilization of their lysosome membrane. However, these functions remain to be evaluated and the question that arises is how are lysosomes in B cells affected by an excess of lipid in LSDs? Thus, alterations in lysosome localization and function could have an impact in B cell activation and its effector functions in NPC disease.

Levels of cytokines and chemokines are increased and participate in the initiation and propagation of the molecular pathogenesis of GD. The excess of glucosylceramide in GD cells can trigger and activate the release of interferon-γ, interleukin 4 and 6, and transforming growth factor-β by macrophages and dendritic cells. This promotes the development of T helper and follicular helper T cells required for the formation and activation of germinal centers that drive B-cell differentiation and thus have an impact on immunoglobulin (IgG, IgA, and IgM) production, triggering hypergammaglobulinemia, which contributes to inflammation (Fazilleau et al., 2009; Pandey and Grabowski 2013; Nguyen et al., 2020). Additionally, the accumulation of lipid rafts and glycosphingolipid storage in B cells in GD and NPC, leads to degradation of lipid raft-associated B cell receptor and thus altered immune responses (Vruchte et al., 2010). In fact, we speculate that it could disrupt BCR-dependent signaling and activation, which can be associated to the decrease in B cell levels observed in GD patients (Limgala et al., 2016). Alternatively, several patients with GD develop neoplasms and altered B-cell proliferation by mechanisms yet to be discovered (Pandey and Grabowski 2013; Cox et al., 2015). Thus, it is important to investigate the contribution of B cell functions and the implication of cathepsins and CD36 under the context of these diseases, where alterations in homeostatic pathways could converge in lysosomal dysfunction and their pathophysiological progress.

## Emerging Cellular Mechanisms in the Control of Lipid Homeostasis: Inter-organelle Contacts

Recent studies have highlighted the importance of organelle contacts in mediating intracellular lipid flux. Compartments such as lysosomes, ER, mitochondria, Golgi complex, and lipid droplets physically interact and communicate with each other, but preserve their compartmentalization without membrane fusion. This form of communication has been denominated Membrane Contact Sites (MCSs), consisting of regions of apposition between two organelles (with a distance between 10 and 30 nm) through anchoring proteins, thus modulating the function of one or both compartments (Ballabio and Bonifacino 2020; Prinz et al., 2020). In recent years, MCSs have gained notorious interest because they are a communication system different from the diffusion of metabolites through membranes and vesicular transport; however, there is still much to be elucidated about the mechanisms that regulate their formation. Nonetheless, among the main functions described for MCSs are signaling between organelles, regulation of membrane dynamics, metabolic channeling, and lipid transport (Prinz et al., 2020). Therefore, alterations in lysosomal homeostasis and function due to lipid accumulation may have far-reaching consequences in communication and cross-regulation between organelles. Interestingly, inter-organelle contacts are involved in the pathogenesis of diseases that present alterations in cholesterol or triglyceride levels, as in obesity, NPC and Gaucher diseases.

### Liver: Inter-organelle Contacts and Lipid Homeostasis in Obesity

Inside the cell, the nutritional context modulates mitochondria-ER membrane contacts, and alterations in this status induce a misbalance in lipid and glucose metabolism (Rieusset 2017). Accordingly, obesity leads to an increase in ER-mitochondrial interactions, resulting in mitochondrial calcium overload, compromised mitochondrial oxidative capacity, and increased oxidative stress, thus accelerating obesity-related pathologies, such as hepatic steatosis and glucose intolerance (Arruda et al., 2014).

Additionally, a recent study showed that the contact between mitochondria and the ER regulates the synthesis of VLDL in response to changes in lipid flux (Anastasia et al., 2021). This was evidenced after observing that hepatic depletion of the ER-resident Microsomal Triglyceride Transfer Protein (MTP), which plays a crucial role in VLDL biogenesis, promotes a phenotype reminiscent of hepatic dyslipidemia, as well as mitochondria wrapped by curved sheets of rough ER increasing the contact regions between them. This alteration reduces VLDL biogenesis and redirects hepatic free FAs flux towards LDs (Kozlitina et al., 2014; Anastasia et al., 2021). This is consistent with what has been described previously, where the accumulation of LDs increases the risk of metabolic disorders such as obesity and insulin resistance (Gross and Silver 2014; Wang et al., 2020b). In this sense, the authors conclude that there is a connection between intracellular and systemic control mechanisms to maintain lipid homeostasis (Anastasia et al., 2021).

**FIGURE 3:**
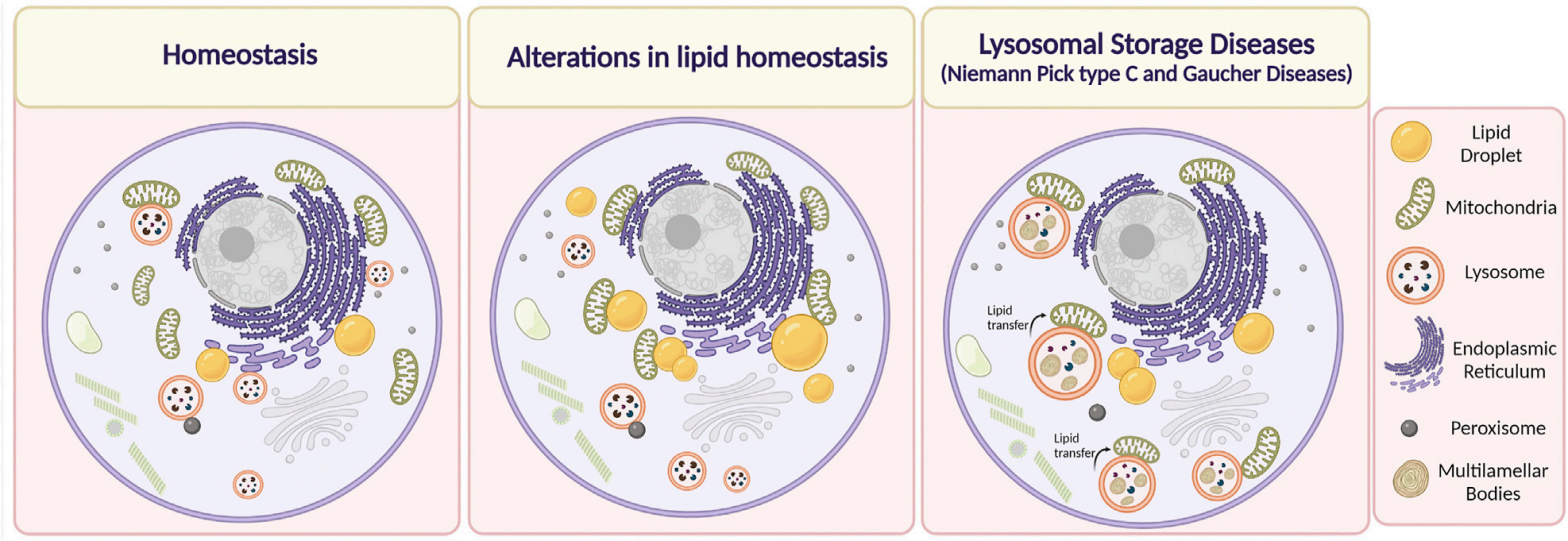
Alterations in Membrane Contact Sites (MCSs) in lipid-related disorders. Under physiological conditions, the organelles are in close contact with each other depending on cell requirements. It has been described that alteration in lipid homeostasis, such as in the case of obesity, dyslipidemias, or after the administration of a high-fat diet induce an increase in MCSs between mitochondria and lipid droplets, and mitochondria and the endoplasmic reticulum. On the other hand, it has been reported that in Niemann Pick type C disease, lysosome-mitochondria MCSs increase, whereas, in the case of Gaucher disease, the duration of these contacts is higher. We speculate that there is an increase in the transfer of lipids between lysosome and mitochondria through MCSs that contributes to mitochondrial damage in NPC and Gaucher cells. Lipids are accumulated inside endolysosomes, in membrane reservoirs (Multilamellar bodies) (Created with BioRender.com).

On the other hand, a relevant type of membrane contact in lipid homeostasis is one formed by LD in tissues highly sensitive to lipid levels such as liver tissue. Interestingly, Krahmer et al. (2018) observed changes in the formation of MCSs in hepatocytes derived from HFD-fed mice and in the proteome associated with LDs. In this context, they found increased mitochondria-LDs contacts and increased binding of proteins belonging to different intracellular organelles (including those involved in MCSs between other organelles) to the surface of LDs. This highlights the tight modulation of metabolic processes by MCSs (Krahmer et al., 2018) (Figure 3).

Overall, these observations reinforce the notion that at least part of the intracellular mechanisms are altered during obesity in tissues that are key to metabolism, resulting from alterations in the communication of intracellular compartments. Considering that MCSs constitute a communication system based on the dynamic, efficient, and rapid transfer of lipids plus other metabolites, these contacts may be part of a central mechanisms underlying alterations in metabolic homeostasis.

### Alterations of Inter-Organelle Contacts Sites in Dysfunctional Lysosomes in NPC and Gaucher Diseases

It is well known that MCSs between the ER and lysosomes are necessary to mediate intracellular homeostasis of cholesterol. Additionally, recent evidence in CHO and HeLa cells has shown that LDL-c can be transferred through contact regions established between the NPC1 transporter and the ER-localized Gramd1b sterol-transporter from late endosome/lysosomes toward the ER (Höglinger et al., 2019). Thus, when large amounts of LDL-c are internalized, a dynamic redistribution of the ER protein Gramd1b contacts NPC1 at lysosomes, promoting lysosomal cholesterol export. Moreover, the authors show that in the absence of functional NPC1, as in NPC disease, this inter-organelle contact is disrupted, contributing to cholesterol accumulation in lysosomes. Interestingly, the authors found that under these conditions, the lysosomes augment contacts with mitochondria. This is relevant because it might constitute a mechanism by which mitochondria raise their cholesterol levels to a pathological state, which can consequently trigger alterations in their function, thus compromising the metabolic state of the cell (Höglinger et al., 2019) (Figure 3).

On the other hand, MCSs between lysosomes and mitochondria are mediated by the lysosomal steroidogenic acute regulatory protein (StAR) D3 (STARD3) protein. This sterol transporter, also located in the lysosomal membrane, has been studied in the context of cholesterol transfer towards ER through MCSs formation (Alpy et al., 2013). Interestingly, we have shown that STARD3 protein levels in NPC were increased in hepatocytes, correlating with elevated cholesterol levels in mitochondria purified from livers of NPC mice, which might contribute to mitochondrial dysfunction (Balboa et al., 2017, 2021).

As mitochondrial dysfunction is observed in NPC disease, the expansion of MCSs between lysosomes and ER have been proposed as a potential strategy for new therapies for this disease (Yu et al., 2005; Kennedy et al., 2014). Interestingly, the number of MCSs between the ER and lysosomes is induced using agents that reduce cholesterol accumulation in NPC disease. For example, the well-known hydroxypropyl-*γ*-cyclodextrin (HPγCD) and hydroxypropyl-β-cyclodextrin (HPβCD) reduce the accumulation of cholesterol in fibroblasts derived from NPC1 patients (Singhal et al., 2018) and promote the association of lysosomes with the ER, without affecting MCSs between lysosomes and mitochondria. As mentioned initially, NPC1 deficiency disturbs autophagic flux, evidenced by the accumulation of autophagic vacuoles (Pacheco and Lieberman 2008). In fact, treatments with cyclodextrin, which enhances autophagy through the activation of TFEB and subsequent induction of lysosomal biogenesis induction, alleviates the intracellular accumulation of free cholesterol (Singhal et al., 2020). Added to this, it should be noted that functional recovery of contact sites has been successfully tested through their artificial expansion. In this context (Hoglinger et al., 2019) used a sterol-insensitive ORP1L mutant (ORP1L is a lysosome-anchored cholesterol sensor), that constitutively binds the protein VAP in the ER membrane, to act as an artificial tether while preventing the transportation of sterols, to expand ER-lysosome MCS. Remarkably, MCSs expansion by overexpression of this artificial tether rescued lysosomal cholesterol accumulation in NPC1-deficient HeLa cells (Höglinger et al., 2019). Similarly, Meneses-Salas et al. (2020) observed a recovery in the percentage of the endosome/lysosome surface in contact with the ER in CHO cells with mutations in the *Npc1* gene*.* This was observed after silencing Annexin A6, a member of the annexin family implicated in the regulation of endo- and exocytic pathways and cholesterol homeostasis by binding to membranes in a calcium-dependent manner (Meneses-Salas et al., 2020). Hence, this evidence reinforces MCSs as functional therapeutic targets.

Concerning GD, there is no available information regarding changes in MCSs formation in hepatocytes and other metabolic tissues. The unique information related to these types of alterations was provided by Kim et al. (2021), which observed that human iPSC-derived dopaminergic neurons were treated with an inhibitor of β-glucocerebrosidase activity (conduritol-b-epoxide) exhibited prolonged tethering between mitochondria-lysosome MCSs (Kim et al., 2021) (Figure 3). Interestingly, in Gaucher and NPC cells there is a secondary accumulation of lipids besides glucosylceramide and cholesterol, respectively. In addition, NPC cells show mitochondrial damage and mitochondrial cholesterol accumulation (Balboa et al., 2017). Hence, it is possible to speculate that there is an increase in the transfer of lipids between lysosome and mitochondria, through MCSs that contributes to mitochondrial damage in NPC and Gaucher cells.

Therefore, the information obtained from the research in LSDs is summarized in the fact that the communication mediated by MCSs between compartments that are relevant for the sensing and modulation of lipid homeostasis, can undergo changes depending on key lysosomal proteins. Although the evidence provided in this area comes from non-metabolic cellular models, it is crucial to address whether similar alterations in contact sites are occurring in cells from metabolic tissues in the context of dyslipidemias. Hence, valuable information can be rescued from the understanding of major networks that regulate the intracellular metabolic state, and with it, of the organism.

### Conclusions and Outstanding Questions

One of the critical organelles that respond to lipids excess are lysosomes. Several studies have conclusively shown that the lysosome-autophagic axis is affected by lipid overload in adipocytes and hepatocytes. Findings discussed in this review show that in an obese state, autophagy is mostly downregulated in adipose tissue and in the liver. Similarly, NPC and Gaucher diseases have an impairment in the clearance of autophagosomes along with proteolytic defects.

We also addressed commonly altered pathways in the lysosome-autophagic axis. On one side, various studies associate lysosomal dysfunction with altered levels of cathepsins. The eventual permeability of lysosomes and the release of cathepsins to the cytosol could lead to mitochondrial damage and promote lysosome dysfunction and cellular death in these tissues, where the overall evidence is not yet conclusive and more studies are needed to clarify how dysregulated cathepsins mediate lysosome defects in obesity, NPC, and Gaucher diseases. On the other hand, studies revealed that CD36 expression levels are increased in obese adipose and liver tissues, which negatively regulates autophagic function and leads to the failure of lysosomal homeostasis. In adipocytes, CD36/Fyn/IP3R1-mediated lysosomal calcium overload can also be associated with a failure in autophagic flux. Additionally, lipid uptake is mediated by an increase in CD36 expression in adipocytes and hepatocytes by PPAR-γ, which promotes lipid accumulation and contributes to lysosome dysfunction. Intriguingly, LPA is critical in lipid metabolism in obesity, and it is possible to speculate that the hepatic PPAR-γ-CD36 pathway is regulated by LPA. Even though upregulation of CD36 expression in NPC liver has been observed, its role in NPC and Gaucher hepatic diseases remains unclear. However, we believe that galectin 3 and LPA may also promote the expression of CD36 in NPC hepatocytes through the PPAR-γ pathway, contributing to lysosome dysfunction probably by analogous mechanisms observed in obesity.

Considering that B cells rely on lysosomes for the extraction and processing of antigens, it is relevant to elucidate how lysosomes and lysosomal hydrolases such as cathepsins, respond to an excess of nutrients in obesity and LSDs, and how these signals crosstalk with the activation of B cells during antigen recognition. Thus, we speculate that homeostatic alterations in CD36 and cathepsins described in obesity in adipocytes and hepatocytes could also be altered in B cells infiltrated in metabolic tissues, promoting functionals changes. Thus, it is essential to understand dysfunctions at this level, given that B cells are mediators of inflammation in adipose and liver tissues.

On the other hand, the studies of MCSs formed by lysosomes have dramatically increased in recent years given the relevance of its impact in lipid metabolism. The evidence suggests that they play a crucial role in the pathogenic mechanisms associated with obesity and its comorbidities, as well as in NPC and Gaucher diseases. In this context, it seems that an increase of MCSs between mitochondria- LDs and mitochondria-ER are also part of the altered cellular mechanisms, reflecting a misbalance in MCSs homeostasis. Thereby, unraveling these potentially disturbed pathways, including mechanisms that regulate MCSs involved in the control of lipid homeostasis will allow us to understand how responses of adipocytes, hepatocytes, and B cells are affected in obesity, NPC and Gaucher diseases. These findings will potentially unmask new key common targets in the modulation of lysosome function for the treatment of disorders related to lipids.

### Outstanding Questions

From the perspective of lysosomal dysfunction observed in obesity, some outstanding questions that remain to be answered in future investigations are:

-How does CD36 coordinate autophagy in adipocytes and hepatocytes? Is there a relationship between the function of CD36 and lysosomal cathepsin activity? What is the implication of CD36 in the uptake of lipids and autophagy in the metabolic tissues of NPC and Gaucher diseases?

-How do LPA and the CD36-PPAR-γ pathway regulate lipid accumulation and lysosome dysfunction in adipocytes and hepatocytes, in obesity? How do LPA and galectin 3 coordinate the activation of this pathway in obesity? How does BMP promote lysosomal dysfunction in pathological states of obesity, NPC and Gaucher?

-Are MCSs altered in a similar fashion by the overload of lipids in adipose tissue and liver in obesity, NPC and Gaucher diseases? How is the formation of MCSs regulated under these conditions? Is there an increased lipid transfer in the transfer of lipids between lysosome and mitochondria through MCSs in LSDs? Does the increased lipid transfer in NPC and Gaucher diseases lead to mitochondrial dysfunction?

**-**With respect to B cells: Does CD36 coordinate lysosomal function in B cells and is it altered during obesity? How do lysosomes in B cells respond to an excess of nutrients in obesity and LSDs?

## Abbreviations

ATX, autotoxin; BCR, B cell receptor; BMP, bis (monoacylglycero) phosphate; CTS, cathepsin; ER, endoplasmic reticulum; FAs, fatty acids; GD, Gaucher Disease; GILT, gamma-interferon-inducible lysosomal thiol reductase; HFD, high-fat diet; LDL, low-density lipoprotein; LDL-c, LDL-cholesterol; LDs, lipid droplets; LPA, lysophosphatidic acid; LSDs, Lysosomal Storage Diseases; MCSs, membrane contact sites; NAFLD, nonalcoholic fatty liver disease; NASH, nonalcoholic steatohepatitis; NPC, Niemann-Pick type C; PPAR-γ, peroxisome proliferator-activated receptor γ; STARD3, steroidogenic acute regulatory protein (StAR) D3; VLDL, low-density lipoprotein; WAT, white adipose tissue.

## References

[B1] Afinogenova Y. Ruan J. Yang R. Kleytman N. Pastores G. Lischuk A. (2019). Aberrant Progranulin, YKL-40, Cathepsin D and Cathepsin S in Gaucher Disease. Mol. Genet. Metab. 128 (1–2), 62–67. 10.1016/j.ymgme.2019.07.014 31358474 PMC6864269

[B2] Alpy F. Rousseau A. Schwab Y. Legueux F. Stoll I. Wendling C. (2013). STARD3/STARD3NL and VAP Make a Novel Molecular Tether between Late Endosomes and the ER. J. Cel Sci. 126 (23), 5500–5512. 10.1242/jcs.139295 24105263

[B3] Amritraj A. Wang Y. Revett T. J. Vergote D. Westaway D. Kar S. (2013). Role of Cathepsin D in U18666A-Induced Neuronal Cell Death. J. Biol. Chem. 288 (5), 3136–3152. 10.1074/jbc.M112.412460 23250759 PMC3561536

[B4] Anastasia I. Ilacqua N. Raimondi A. Lemieux P. Ghandehari-Alavijeh R. Faure G. (2021). Mitochondria-Rough-ER Contacts in the Liver Regulate Systemic Lipid Homeostasis. Cel Rep. 34 (11), 108873. 10.1016/j.celrep.2021.108873 33730569

[B5] Appelqvist H. Nilsson C. Garner B. Brown A. J. Kågedal K. Öllinger K. (2011). Attenuation of the Lysosomal Death Pathway by Lysosomal Cholesterol Accumulation. Am. J. Pathol. 178 (2), 629–639. 10.1016/j.ajpath.2010.10.030 21281795 PMC3069902

[B6] Appelqvist H. Wäster P. Kågedal K. Öllinger K. (2013). The Lysosome: From Waste Bag to Potential Therapeutic Target. J. Mol. Cel Biol. 5 (4), 214–226. 10.1093/jmcb/mjt022 23918283

[B7] Arbogast F. Arnold J. Hammann P. Kuhn L. Chicher J. Murera D. (2019). ATG5 Is Required for B Cell Polarization and Presentation of Particulate Antigens. Autophagy 15 (2), 280–294. 10.1080/15548627.2018.1516327 30196744 PMC6333460

[B8] Arruda A. P. Pers B. M. Parlakgül G. Güney E. Inouye K. Hotamisligil G. S. (2014). Chronic Enrichment of Hepatic Endoplasmic Reticulum-Mitochondria Contact Leads to Mitochondrial Dysfunction in Obesity. Nat. Med. 20 (12), 1427–1435. 10.1038/nm.3735 25419710 PMC4412031

[B9] Aschermann S. Lux A. Baerenwaldt A. Biburger M. Nimmerjahn F. (2009). The Other Side of Immunoglobulin G: Suppressor of Inflammation. Clin. Exp. Immunol. 160 (2), 161–167. 10.1111/j.1365-2249.2009.04081.x 20041883 PMC2857938

[B10] Balboa E. Castro J. Pinochet M.-J. Cancino G. I. Matías N. Sáez P. J. (2017). MLN64 Induces Mitochondrial Dysfunction Associated with Increased Mitochondrial Cholesterol Content. Redox Biol. 12 (February), 274–284. 10.1016/j.redox.2017.02.024, 28282615 PMC5344325

[B11] Balboa E. Marín T. Oyarzún J. E. Contreras P. S. Hardt R. Van Den Bosch T. (2021). Proteomic Analysis of Niemann-Pick Type C Hepatocytes Reveals Potential Therapeutic Targets for Liver Damage. Cells 10, 2159. 10.3390/cells10082159 34440927 PMC8392304

[B12] Ballabio A. Bonifacino J. S. (2020). Lysosomes as Dynamic Regulators of Cell and Organismal Homeostasis. Nat. Rev. Mol. Cel Biol. 21 (2), 101–118. 10.1038/s41580-019-0185-4 31768005

[B13] Bechmann L. P. Gieseler R. K. Sowa J.-P. Kahraman A. Erhard J. Wedemeyer I. (2010). Apoptosis Is Associated with CD36/Fatty Acid Translocase Upregulation in Non-alcoholic Steatohepatitis. Liver Int. 30 (6), 850–859. 10.1111/j.1478-3231.2010.02248.x 20408954

[B14] Beltroy E. P. Liu B. Dietschy J. M. Turley S. D. (2007). Lysosomal Unesterified Cholesterol Content Correlates with Liver Cell Death in Murine Niemann-Pick Type C Disease. J. Lipid Res. 48 (4), 869–881. 10.1194/jlr.M600488-JLR200 17220530

[B15] Beltroy E. P. Richardson J. A. Horton J. D. Turley S. D. Dietschy J. M. (2005). Cholesterol Accumulation and Liver Cell Death in Mice with Niemann-Pick Type C Disease. Hepatology 42 (4), 886–893. 10.1002/hep.20868 16175610

[B16] Blüher M. (2019). Obesity: Global Epidemiology and Pathogenesis. Nat. Rev. Endocrinol. 15 (5), 288–298. 10.1038/s41574-019-0176-8 30814686

[B17] Blum J. S. Wearsch P. A. Cresswell P. (2013). Pathways of Antigen Processing. Annu. Rev. Immunol. 31 (1), 443–473. 10.1146/annurev-immunol-032712-095910 23298205 PMC4026165

[B18] Bournat J. C. Brown. C. W. (2010). Mitochondrial Dysfunction in Obesity. Curr. Opin. Endocrinol. Diabetes Obes. 17 (5), 446–452. 10.1016/j.lfs.2017.11.01910.1097/med.0b013e32833c3026 20585248 PMC5001554

[B19] Brown R. A. Voit A. Srikanth M. P. Thayer J. A. Kingsbury T. J. Jacobson M. A. (2019). mTOR Hyperactivity Mediates Lysosomal Dysfunction in Gaucher's Disease iPSC-Neuronal Cells. Dis. Models Mech. 12 (10). 10.1242/dmm.038596 PMC6826018 31519738

[B20] Buqué X. Cano A. Miquilena-Colina M. E. García-Monzón C. Ochoa B. Aspichueta P. (2012). High Insulin Levels Are Required for FAT/CD36 Plasma Membrane Translocation and Enhanced Fatty Acid Uptake in Obese Zucker Rat Hepatocytes. Am. J. Physiology-endocrinology Metab. 303 (4), E504–E514. 10.1152/ajpendo.00653.2011 22693206

[B21] Byeon S. K. Lee J. Y. Lee J.-S. Moon M. H. (2015). Lipidomic Profiling of Plasma and Urine from Patients with Gaucher Disease during Enzyme Replacement Therapy by Nanoflow Liquid Chromatography-Tandem Mass Spectrometry. J. Chromatogr. A. 1381, 132–139. 10.1016/j.chroma.2015.01.004 25597892

[B22] Cai L. Wang Z. Ji A. Meyer J. M. van der Westhuyzen D. R. (2012). Scavenger Receptor CD36 Expression Contributes to Adipose Tissue Inflammation and Cell Death in Diet-Induced Obesity. PLoS ONE 7 (5), e36785. 10.1371/journal.pone.0036785 22615812 PMC3353961

[B23] Castaneda J. A. Lim M. J. Cooper J. D. Pearce D. A. (2008). Immune System Irregularities in Lysosomal Storage Disorders. Acta Neuropathol. 115 (2), 159–174. 10.1007/s00401-007-0296-4 17924126

[B24] Cermak S. Kosicek M. Mladenovic-Djordjevic A. Smiljanic K. Kanazir S. Hecimovic S. (2016). “Loss of Cathepsin B and L Leads to Lysosomal Dysfunction, NPC-like Cholesterol Sequestration and Accumulation of the Key Alzheimer's Proteins,”. Editor Lakshmana M. K., 11, e0167428. 10.1371/journal.pone.0167428 PLOS ONE 11 27902765 PMC5130271

[B25] Chait A. den Hartigh L. J. (2020). Adipose Tissue Distribution, Inflammation and its Metabolic Consequences, Including Diabetes and Cardiovascular Disease. Front. Cardiovasc. Med. 7 (February), 1–41. 10.3389/fcvm.2020.00022 32158768 PMC7052117

[B26] Christian P. Sacco J. Adeli K. (2013). Autophagy: Emerging Roles in Lipid Homeostasis and Metabolic Control. Biochim. Biophys. Acta (Bba) - Mol. Cel Biol. Lipids 1831 (4), 819–824. 10.1016/j.bbalip.2012.12.009 23274236

[B27] Chung C. Puthanveetil P. Ory D. S. Lieberman A. P. (2016). Genetic and Pharmacological Evidence Implicates Cathepsins in Niemann-Pick C Cerebellar Degeneration. Hum. Mol. Genet. 25 (7), 1434–1446. 10.1093/hmg/ddw025 26908626 PMC4787909

[B28] Cleeter M. W. J. Chau K.-Y. Gluck C. Mehta A. Hughes D. A. Duchen M. (2013). Glucocerebrosidase Inhibition Causes Mitochondrial Dysfunction and Free Radical Damage. Neurochem. Int. 62 (1), 1–7. 10.1016/j.neuint.2012.10.010 23099359 PMC3550523

[B29] Clemente-Postigo M. Tinahones A. El Bekay R. Malagón M. M. Tinahones F. J. (2020). The Role of Autophagy in White Adipose Tissue Function: Implications for Metabolic Health. Metabolites 10 (5), 179. 10.3390/metabo10050179 PMC7281383 32365782

[B30] Cluzeau C. V. M. Watkins-Chow D. E. Fu R. Borate B. Yanjanin N. Dail M. K. (2012). Microarray Expression Analysis and Identification of Serum Biomarkers for Niemann-Pick Disease, Type C1. Hum. Mol. Genet. 21 (16), 3632–3646. 10.1093/hmg/dds193 22619379 PMC3406758

[B31] Corcoran L. Vremec D. Febbraio M. Baldwin T. Handman E. (2002). Differential Regulation of CD36 Expression in Antigen-Presenting Cells: Oct-2 Dependence in B Lymphocytes but Not Dendritic Cells or Macrophages. Int. Immunol. 14 (10), 1099–1104. 10.1093/intimm/dxf075 12356675

[B32] Cousin B. a. Andr M. Casteilla L. Pnicaud L. (2001). Altered Macrophage-like Functions of Preadipocytes in Inflammation and Genetic Obesity. J. Cel. Physiol. 186 (3), 380–386. 10.1002/1097-4652(2001)9999:9999<000:aid-jcp1038>3.0.co;2-t 11169977

[B33] Couturier J. Nuotio-Antar A. M. Agarwal N. Wilkerson G. K. Saha P. Kulkarni V. (2019). Lymphocytes Upregulate CD36 in Adipose Tissue and Liver. Adipocyte 8 (1), 154–163. 10.1080/21623945.2019.1609202 31035848 PMC6768236

[B34] Cox T. M. Rosenbloom B. E. Barker R. A. (2015). Gaucher Disease and Comorbidities: B-Cell Malignancy and Parkinsonism. Am. J. Hematol. 90 (S1), S25–S28. 10.1002/ajh.24057 26096744

[B35] Dai S. Dulcey A. E. Hu X. Wassif C. A. Porter F. D. Austin C. P. (2017). Methyl-β-cyclodextrin Restores Impaired Autophagy Flux in Niemann-Pick C1-Deficient Cells through Activation of AMPK. Autophagy 13 (8), 1435–1451. 10.1080/15548627.2017.1329081 28613987 PMC5584846

[B36] Davidson C. D. Ali N. F. Micsenyi M. C. Stephney G. Renault S. Dobrenis K. (2009). Chronic Cyclodextrin Treatment of Murine Niemann-Pick C Disease Ameliorates Neuronal Cholesterol and Glycosphingolipid Storage and Disease Progression. PLoS ONE 4 (9), e6951. 10.1371/journal.pone.0006951 19750228 PMC2736622

[B37] Davis O. B. Shin H. R. Lim C.-Y. Wu E. Y. Kukurugya M. Maher C. F. 2021. “NPC1-MTORC1 Signaling Couples Cholesterol Sensing to Organelle Homeostasis and Is a Targetable Pathway in Niemann-Pick Type C.” Developmental Cel 56(3):260–276.e7. 10.1016/j.devcel.2020.11.016 PMC8919971 33308480

[B38] Droga-Mazovec G. Bojič L. Petelin A. Ivanova S. Romih R. Repnik U. (2008). Cysteine Cathepsins Trigger Caspase-dependent Cell Death through Cleavage of Bid and Antiapoptotic Bcl-2 Homologues. J. Biol. Chem. 283 (27), 19140–19150. 10.1074/jbc.M802513200 18469004

[B39] D’Souza K. Paramel G. Kienesberger P. (2018). Lysophosphatidic Acid Signaling in Obesity and Insulin Resistance. Nutrients 10 (4), 399. 10.3390/nu10040399 PMC5946184 29570618

[B40] Dugail I. (2014). Lysosome/Lipid Droplet Interplay in Metabolic Diseases. Biochimie 96 (1), 102–105. 10.1016/j.biochi.2013.07.008 23880642

[B41] Dusaulcy R. Rancoule C. Grès S. Wanecq E. Colom A. Guigné C. (2011). Adipose-Specific Disruption of Autotaxin Enhances Nutritional Fattening and Reduces Plasma Lysophosphatidic Acid. J. Lipid Res. 52 (6), 1247–1255. 10.1194/jlr.M014985 21421848 PMC3090245

[B42] Ejarque M. Ceperuelo-Mallafré V. Serena C. Maymo-Masip E. Duran X. Díaz-Ramos Monica. (2019). Adipose Tissue Mitochondrial Dysfunction in Human Obesity Is Linked to a Specific DNA Methylation Signature in Adipose-Derived Stem Cells. Int. J. Obes. 43 (6), 1256–1268. 10.1038/s41366-018-0219-6 PMC6760577 30262812

[B43] Elrick M. J. Yu T. Chung C. Lieberman A. P. (2012). Impaired Proteolysis Underlies Autophagic Dysfunction in Niemann-Pick Type C Disease. Hum. Mol. Genet. 21 (22), 4876–4887. 10.1093/hmg/dds324 22872701 PMC3607480

[B44] Engin A. B. Basak Ayse. (2017). “What Is Lipotoxicity,” in Advances in Experimental Medicine and Biology. Editors Engin A. B. Engin A. (Cham: Springer International Publishing), 197–220. 10.1007/978-3-319-48382-5_8

[B45] Fan Y. Yang J. Li H. Li H. Zhang S. Li X. (2020). SNX10 Deficiency Restricts Foam Cell Formation and Protects against Atherosclerosis by Suppressing CD36-Lyn Axis. Can. J. Cardiol. 10.1016/j.cjca.2020.05.010 32428616

[B46] Farfel-Becker T. Vitner E. B. Kelly S. L. Bame J. R. Duan J. Shinder V. (2014). Neuronal Accumulation of Glucosylceramide in a Mouse Model of Neuronopathic Gaucher Disease Leads to Neurodegeneration. Hum. Mol. Genet. 23 (4), 843–854. 10.1093/hmg/ddt468 24064337 PMC3900102

[B47] Fazilleau N. Mark L. McHeyzer-Williams L. J. McHeyzer-Williams M. G. (2009). Follicular Helper T Cells: Lineage and Location. Immunity 30 (3), 324–335. 10.1016/j.immuni.2009.03.003 19303387 PMC2731675

[B48] Febbraio M. Silverstein R. L. (2007). CD36: Implications in Cardiovascular Disease. Int. J. Biochem. Cel Biol. 39 (11), 2012–2030. 10.1016/j.biocel.2007.03.012 PMC2034445 17466567

[B49] Feldstein A. E. Werneburg N. W. Li Z. Bronk S. F. Gores G. J. (2006). Bax Inhibition Protects against Free Fatty Acid-Induced Lysosomal Permeabilization. Am. J. Physiology-gastrointestinal Liver Physiol. 290 (6), G1339–G1346. 10.1152/ajpgi.00509.2005 PMC3056273 16484678

[B50] Ferry G. Tellier E. Try A. Grés S. Naime I. Simon M. F. (2003). Autotaxin Is Released from Adipocytes, Catalyzes Lysophosphatidic Acid Synthesis, and Activates Preadipocyte Proliferation. J. Biol. Chem. 278 (20), 18162–18169. 10.1074/jbc.M301158200 12642576 PMC1885458

[B51] Fucho R. Martínez L. Baulies A. Torres S. Tarrats N. Fernandez A. (2014). ASMase Regulates Autophagy and Lysosomal Membrane Permeabilization and its Inhibition Prevents Early Stage Non-alcoholic Steatohepatitis. J. Hepatol. 61 (5), 1126–1134. 10.1016/j.jhep.2014.06.009 24946279 PMC4203709

[B52] Gaidhu M. P. Anthony N. M. Patel P. Hawke T. J. Ceddia R. B. (2010). Dysregulation of Lipolysis and Lipid Metabolism in Visceral and Subcutaneous Adipocytes by High-Fat Diet: Role of ATGL, HSL, and AMPK. Am. J. Physiology-cell Physiol. 298 (4), C961–C971. 10.1152/ajpcell.00547.2009 20107043

[B53] Gallala H. D. Sandhoff K. (2011). Biological Function of the Cellular Lipid BMP-BMP as a Key Activator for Cholesterol Sorting and Membrane Digestion. Neurochem. Res. 36 (9), 1594–1600. 10.1007/s11064-010-0337-6 21136156

[B54] García-Barrado M. Iglesias-Osma M. Pérez-García E. Carrero S. Blanco E. Carretero-Hernández M. (2020). Role of Flavonoids in the Interactions Among Obesity, Inflammation, and Autophagy. Pharmaceuticals 13 (11), 342. 10.3390/ph13110342 PMC7692407 33114725

[B55] García-Sanz P. Orgaz L. Bueno-Gil G. Espadas I. Rodríguez-Traver E. Kulisevsky J. (2017). N370S -GBA1 Mutation Causes Lysosomal Cholesterol Accumulation in Parkinson’s Disease. Mov Disord. 32 (10), 1409–1422. 10.1002/mds.27119 28779532

[B56] Gillotte-Taylor K. Boullier A. Witztum J. L. Steinberg D. Quehenberger O. (2001). Scavenger Receptor Class B Type I as a Receptor for Oxidized Low Density Lipoprotein. J. Lipid Res. 42 (9), 1474–1482. PMID: 11518768. 10.1016/s0022-2275(20)30281-9 11518768

[B57] Gornicka A. Fettig J. Eguchi A. Berk M. P. Thapaliya S. Dixon L. J. (2012). Adipocyte Hypertrophy Is Associated with Lysosomal Permeability Both *In Vivo* and *In Vitro*: Role in Adipose Tissue Inflammation. Am. J. Physiology-endocrinology Metab. 303 (5), E597–E606. 10.1152/ajpendo.00022.2012 PMC3468510 22739104

[B58] Grabner G. F. Fawzy N. Pribasnig M. A. Trieb M. Taschler U. Holzer M. (2019). Metabolic Disease and ABHD6 Alter the Circulating Bis(Monoacylglycerol)Phosphate Profile in Mice and Humans. J. Lipid Res. 60 (5), 1020–1031. 10.1194/jlr.M093351 30894461 PMC6495172

[B59] Gross D. A. Silver D. L. (2014). Cytosolic Lipid Droplets: From Mechanisms of Fat Storage to Disease. Crit. Rev. Biochem. Mol. Biol. 49 (4), 304–326. 10.3109/10409238.2014.931337 25039762

[B60] Hao J.-W. Wang J. Guo H. Zhao Y.-Y. SunZhao H.-H. Li Y.-F. (2020). CD36 Facilitates Fatty Acid Uptake by Dynamic Palmitoylation-Regulated Endocytosis. Nat. Commun. 11 (1), 1–16. 10.1038/s41467-020-18565-8 32958780 PMC7505845

[B61] Harwood N. E. Batista F. D. (2010). Early Events in B Cell Activation. Annu. Rev. Immunol. 28 (1), 185–210. 10.1146/annurev-immunol-030409-101216 20192804

[B62] He C. Wang S. Zhou C. He M. Wang J. Ladds M. (2021). CD36 and LC3B Initiated Autophagy in B Cells Regulates the Humoral Immune Response. Autophagy 00 (00), 1–15. 10.1080/15548627.2021.1885183 PMC8632284 33535890

[B63] Helkin A. Stein J. J. Lin S. Siddiqui S. Maier K. G. Gahtan V. (2016). Dyslipidemia Part 1-Review of Lipid Metabolism and Vascular Cell Physiology. Vasc. Endovascular Surg. 50 (2), 107–118. 10.1177/1538574416628654 26983667

[B64] Höglinger D. Burgoyne T. Sanchez-Heras E. Hartwig P. Colaco A. Newton J. (2019). NPC1 Regulates ER Contacts with Endocytic Organelles to Mediate Cholesterol Egress. Nat. Commun. 10 (1), 1–14. 10.1038/s41467-019-12152-2 31537798 PMC6753064

[B65] Honey K. Duff M. Beers C. Brissette W. H. Elliott E. A. Peters C. (2001). Cathepsin S Regulates the Expression of Cathepsin L and the Turnover of γ-Interferon-Inducible Lysosomal Thiol Reductase in B Lymphocytes. J. Biol. Chem. 276 (25), 22573–22578. 10.1074/jbc.m101851200 11306582

[B66] Iacobini C. Menini S. Ricci C. Fantauzzi C. B. Scipioni A. Salvi L. (2011). Galectin-3 Ablation Protects Mice from Diet-Induced NASH: A Major Scavenging Role for Galectin-3 in Liver. J. Hepatol. 54 (5), 975–983. 10.1016/j.jhep.2010.09.020 21145823

[B67] Ilnytska O. Jeziorek M. Lai K. Altan-Bonnet N. Dobrowolski R. Storch J. (2021). Lysobisphosphatidic Acid (LBPA) Enrichment Promotes Cholesterol Egress via Exosomes in Niemann Pick Type C1 Deficient Cells. Biochim. Biophys. Acta (Bba) - Mol. Cel Biol. Lipids 1866 (6), 158916. 10.1016/j.bbalip.2021.158916 PMC8038758 33716137

[B68] Inami Y. Yamashina S. Izumi K. Ueno T. Tanida I. Ikejima K. (2011). Hepatic Steatosis Inhibits Autophagic Proteolysis via Impairment of Autophagosomal Acidification and Cathepsin Expression. Biochem. Biophysical Res. Commun. 412 (4), 618–625. 10.1074/jbc.M10185120010.1016/j.bbrc.2011.08.012 21856284

[B69] Ireland J. M. Unanue E. R. (2011). Autophagy in Antigen-Presenting Cells Results in Presentation of Citrullinated Peptides to CD4 T Cells. J. Exp. Med. 208 (13), 2625–2632. 10.1084/jem.20110640 22162830 PMC3244027

[B70] Ivanova M. M. Changsila E. Iaonou C. Goker-Alpan O. (2019). Impaired Autophagic and Mitochondrial Functions Are Partially Restored by ERT in Gaucher and Fabry Diseases. PLoS ONE 14 (1), e0210617–22. 10.1371/journal.pone.0210617 30633777 PMC6329517

[B71] Jaishy B. Abel E. D. (2016). Lipids, Lysosomes, and Autophagy. J. Lipid Res. 57 (9), 1619–1635. 10.1194/jlr.R067520 27330054 PMC5003162

[B72] Jakab J. Miškić B. Mikšić Š. Juranić B. Ćosić V. Schwarz D. (2021). Adipogenesis as a Potential Anti-obesity Target: A Review of Pharmacological Treatment and Natural Products. Dmso 14, 67–83. 10.2147/DMSO.S281186 PMC7802907 33447066

[B73] Jose A. Kienesberger P. C. (2021). Autotaxin-Lpa-Lpp3 Axis in Energy Metabolism and Metabolic Disease. Ijms 22 (17), 9575. 10.3390/ijms22179575 34502491 PMC8431043

[B74] Ju L. Han J. Zhang X. Deng Y. Yan H. Wang C. (2019). Obesity-associated Inflammation Triggers an Autophagy-Lysosomal Response in Adipocytes and Causes Degradation of Perilipin 1. Cell Death Dis 10 (2), 121. 10.1038/s41419-019-1393-8 30741926 PMC6370809

[B75] Jung U. Choi M.-S. (2014). Obesity and its Metabolic Complications: The Role of Adipokines and the Relationship between Obesity, Inflammation, Insulin Resistance, Dyslipidemia and Nonalcoholic Fatty Liver Disease. Ijms 15 (4), 6184–6223. 10.3390/ijms15046184 24733068 PMC4013623

[B76] Kaffe E. Magkrioti C. Aidinis V. (2019). Deregulated Lysophosphatidic Acid Metabolism and Signaling in Liver Cancer. Cancers 11 (11), 1626. 10.3390/cancers11111626 PMC6893780 31652837

[B77] Kahn B. B. Flier J. S. (2000). Obesity and Insulin Resistance. J. Clin. Invest. 106 (4), 473–481. 10.1172/JCI10842 10953022 PMC380258

[B78] Kałużna M. Trzeciak I. Ziemnicka K. Machaczka M. Ruchała M. (2019). Endocrine and Metabolic Disorders in Patients with Gaucher Disease Type 1: A Review. Orphanet J. Rare Dis. 14 (1), 1–14. 10.1186/s13023-019-1211-5 31791361 PMC6889605

[B79] Kaminskyy V. Zhivotovsky B. (2012). Proteases in Autophagy. Biochim. Biophys. Acta (Bba) - Proteins Proteomics 1824 (1), 44–50. 10.1016/j.bbapap.2011.05.013 21640203

[B80] Kao D. Danzer H. Collin M. Groß A. Eichler J. Stambuk J. (2015). A Monosaccharide Residue Is Sufficient to Maintain Mouse and Human IgG Subclass Activity and Directs IgG Effector Functions to Cellular Fc Receptors. Cel Rep. 13 (11), 2376–2385. 10.1016/j.celrep.2015.11.027 26670049

[B81] Kennedy B. E. Madreiter C. T. Vishnu N. Malli R. Graier W. F. Karten B. (2014). Adaptations of Energy Metabolism Associated with Increased Levels of Mitochondrial Cholesterol in Niemann-Pick Type C1-Deficient Cells. J. Biol. Chem. 289 (23), 16278–16289. 10.1074/jbc.M114.559914 24790103 PMC4047397

[B82] Kennedy D. J. Kuchibhotla S. Westfall K. M. Westfall Roy. L. Silverstein R. L. Morton R. E. (2011). A CD36-dependent Pathway Enhances Macrophage and Adipose Tissue Inflammation and Impairs Insulin Signalling. Cardiovasc. Res. 89 (3), 604–613. 10.1093/cvr/cvq360 21088116 PMC3028977

[B83] Khan S. Chan Y. T. Revelo X. S. Winer D. A. (2020). The Immune Landscape of Visceral Adipose Tissue during Obesity and Aging. Front. Endocrinol. 11, 1–18. 10.3389/fendo.2020.00267 PMC7243349 32499756

[B84] Kim S. Wong Y. C. Gao F. Krainc D. (2021). Dysregulation of Mitochondria-Lysosome Contacts by GBA1 Dysfunction in Dopaminergic Neuronal Models of Parkinson's Disease. Nat. Commun. 12 (1). 10.1038/s41467-021-22113-3 PMC7985376 33753743

[B85] Klop B. Elte J. Cabezas M. (2013). Dyslipidemia in Obesity: Mechanisms and Potential Targets. Nutrients 5 (4), 1218–1240. 10.3390/nu5041218 23584084 PMC3705344

[B86] Koga H. Kaushik S. Cuervo A. M. (2010). Altered Lipid Content Inhibits Autophagic Vesicular Fusion. FASEB j. 24 (8), 3052–3065. 10.1096/fj.09-144519 20375270 PMC2909278

[B87] Koonen D. P. Y. Jacobs R. L. Febbraio M. Young M. E. Soltys C.-L. M. Ong H. (2007). Increased Hepatic CD36 Expression Contributes to Dyslipidemia Associated with Diet-Induced Obesity. Diabetes 56 (12), 2863–2871. 10.2337/db07-0907 17728375

[B88] Kounakis K. Chaniotakis M. Markaki M. Tavernarakis N. (2019). Emerging Roles of Lipophagy in Health and Disease. Front. Cel Dev. Biol. 7 (SEP), 1–8. 10.3389/fcell.2019.00185 PMC6746960 31552248

[B89] Kozlitina J. Smagris E. Stender S. Nordestgaard B. G. Zhou H. H. Tybjærg-Hansen A. (2014). Exome-Wide Association Study Identifies a TM6SF2 Variant that Confers Susceptibility to Nonalcoholic Fatty Liver Disease. Nat. Genet. 46 (4), 352–356. 10.1038/ng.2901 24531328 PMC3969786

[B90] Krahmer N. Najafi B. Schueder F. Quagliarini F. Steger M. Seitz S. (2018). Organellar Proteomics and Phospho-Proteomics Reveal Subcellular Reorganization in Diet-Induced Hepatic Steatosis. Developmental Cel 47 (2), 205–221. e7. 10.1016/j.devcel.2018.09.017 30352176

[B91] Kulinski A. Vance J. E. (2007). Lipid Homeostasis and Lipoprotein Secretion in Niemann-Pick C1-Deficient Hepatocytes. J. Biol. Chem. 282 (3), 1627–1637. 10.1074/jbc.M610001200 17107950

[B92] Lachmann R. H. Te Vruchte D. Lloyd-Evans E. Reinkensmeier G. Sillence D. J. Fernandez-Guillen L. (2004). Treatment with Miglustat Reverses the Lipid-Trafficking Defect in Niemann-Pick Disease Type C. Neurobiol. Dis. 16 (3), 654–658. 10.1016/j.nbd.2004.05.002 15262277

[B93] Lahiri V. Hawkins W. D. Klionsky D. J. (2019). Watch what You (Self-) Eat: Autophagic Mechanisms that Modulate Metabolism. Cel Metab. 29 (4), 803–826. 10.1016/j.cmet.2019.03.003 PMC6450419 30943392

[B94] Lankar D. Vincent-Schneider H. Briken V. Yokozeki T. Raposo G. Bonnerot C. (2002). Dynamics of Major Histocompatibility Complex Class II Compartments during B Cell Receptor-Mediated Cell Activation. J. Exp. Med. 195 (4), 461–472. 10.1084/jem.20011543 11854359 PMC2193618

[B95] Lanzavecchia A. (1985). Antigen-Specific Interaction between T and B Cells. 1985. J. Immunol. 179 (11), 7206–7208. 18025160

[B96] Lee H. Lee I. S. Choue R. (2013). Obesity, Inflammation and Diet. Pediatr. Gastroenterol. Hepatol. Nutr. 16 (3), 143. 10.5223/pghn.2013.16.3.143 24224147 PMC3819692

[B97] Lee J. H. Zhou J. Xie W. (2008). PXR and LXR in Hepatic Steatosis: A New Dog and an Old Dog with New Tricks. Mol. Pharmaceutics 5 (1), 60–66. 10.1021/mp700121u 18072748

[B98] Li Y. Chao X. Yang L. Lu Q. Li T. Ding W.-X. (2018). Impaired Fasting-Induced Adaptive Lipid Droplet Biogenesis in Liver-specific Atg5-Deficient Mouse Liver Is Mediated by Persistent Nuclear Factor-like 2 Activation. Am. J. Pathol. 188 (8), 1833–1846. 10.1016/j.ajpath.2018.04.015 29803835 PMC6099336

[B99] Li Y. Yang P. Zhao L. Chen Y. Zhang X. Zeng S. (2019). CD36 Plays a Negative Role in the Regulation of Lipophagy in Hepatocytes through an AMPK-dependent Pathway. J. Lipid Res. 60 (4), 844–855. 10.1194/jlr.M090969 30662007 PMC6446711

[B100] Limgala R. P. Ioanou C. Plassmeyer M. Ryherd M. Kozhaya L. Austin L. (2016). Time of Initiating Enzyme Replacement Therapy Affects Immune Abnormalities and Disease Severity in Patients with Gaucher Disease. PLoS ONE 11 (12), e0168135–16. 10.1371/journal.pone.0168135 27942037 PMC5152900

[B101] Lin Y. Cai X. Wang G. Ouyang G. Cao H. (2018). Model Construction of Niemann-Pick Type C Disease in Zebrafish. Biol. Chem. 399 (8), 903–910. 10.1515/hsz-2018-0118 29897878

[B102] Liu B. Turley S. D. Burns D. K. Miller A. M. Repa J. J. Dietschy J. M. (2009). Reversal of Defective Lysosomal Transport in NPC Disease Ameliorates Liver Dysfunction and Neurodegeneration in the Npc1-/- Mouse. Proc. Natl. Acad. Sci. 106 (7), 2377–2382. 10.1073/pnas.0810895106 19171898 PMC2650164

[B103] Liu B. Xie C. Richardson J. A. Turley S. D. Dietschy J. M. (2007). Receptor-Mediated and Bulk-phase Endocytosis Cause Macrophage and Cholesterol Accumulation in Niemann-Pick C Disease. J. Lipid Res. 48 (8), 1710–1723. 10.1194/jlr.M700125-JLR200 17476031

[B104] Liu N. Tengstrand E. A. Chourb L. Hsieh F. Y. (2014). Di-22:6-Bis(Monoacylglycerol)Phosphate: A Clinical Biomarker of Drug-Induced Phospholipidosis for Drug Development and Safety Assessment. Toxicol. Appl. Pharmacol. 279 (3), 467–476. 10.1016/j.taap.2014.06.014 24967688

[B105] Liu R. Nikolajczyk B. S. (2019). Tissue Immune Cells Fuel Obesity-Associated Inflammation in Adipose Tissue and beyond. Front. Immunol. 10. 10.3389/fimmu.2019.01587 PMC6653202 31379820

[B106] Love-Gregory L. Abumrad N. A. (2011). CD36 Genetics and the Metabolic Complications of Obesity. Curr. Opin. Clin. Nutr. Metab. Care 14 (6), 527–534. 10.1097/MCO.0b013e32834bbac9 21912245 PMC3295590

[B107] Luo X. Li Y. Yang P. Chen Y. Wei L. Yu T. (2020). Obesity Induces Preadipocyte CD36 Expression Promoting Inflammation via the Disruption of Lysosomal Calcium Homeostasis and Lysosome Function. EBioMedicine 56, 102797. 10.1016/j.ebiom.2020.102797 32516742 PMC7281849

[B108] Maetzel D. Sarkar S. Wang H. Abi-Mosleh L. Xu P. Cheng A. W. (2014). Genetic and Chemical Correction of Cholesterol Accumulation and Impaired Autophagy in Hepatic and Neural Cells Derived from Niemann-Pick Type C Patient-specific IPS Cells. Stem Cel Rep. 2 (6), 866–880. 10.1016/j.stemcr.2014.03.014 PMC4050353 24936472

[B109] Magro dos Reis I. Houben T. Oligschläger Y. Bücken L. Steinbusch H. Cassiman D. (2020). Dietary Plant Stanol Ester Supplementation Reduces Peripheral Symptoms in a Mouse Model of Niemann-Pick Type C1 Disease. J. Lipid Res. 61 (6), 830–839. 10.1194/jlr.RA120000632 32291331 PMC7269767

[B110] Marques A. R. A. Saftig P. (2019). Lysosomal Storage Disorders - Challenges, Concepts and Avenues for Therapy: beyond Rare Diseases. J. Cel Sci. 132 (2), jcs221739. 10.1242/jcs.221739 30651381

[B111] Martinez-Lopez N. Singh R. (2015). Autophagy and Lipid Droplets in the Liver. Annu. Rev. Nutr. 35 (1), 215–237. 10.1146/annurev-nutr-071813-105336 26076903 PMC7909712

[B112] Martinez-Martin N. Maldonado P. Gasparrini F. Frederico B. Aggarwal S. Gaya M. (2017). A Switch from Canonical to Noncanonical Autophagy Shapes B Cell Responses. Science 355 (6325), 641–647. 10.1126/science.aal3908 28183981 PMC5805088

[B114] McCann M. R. Ratneswaran A. (2019). The Role of PPARγ in Childhood Obesity-Induced Fractures. Genes Nutr. 14 (1), 31. 10.1186/s12263-019-0653-7 31798753 PMC6880598

[B115] McCauliff L. A. Langan A. Li R. Ilnytska O. Bose D. Waghalter M. (2019). Intracellular Cholesterol Trafficking Is Dependent upon NPC2 Interaction with Lysobisphosphatidic Acid. ELife 8, 1–31. 10.7554/eLife.50832 PMC6855803 31580258

[B116] McGrath M. E. (1999). The Lysosomal Cysteine Proteases. Annu. Rev. Biophys. Biomol. Struct. 28 (1), 181–204. 10.1146/annurev.biophys.28.1.181 10410800

[B117] McIntyre T. M. Pontsler A. V. Hilaire St. Silva A. R. St. Hilaire A. Xu Y. (2003). Identification of an Intracellular Receptor for Lysophosphatidic Acid (LPA): LPA Is a Transcellular PPAR Agonist. Proc. Natl. Acad. Sci. 100 (1), 131–136. 10.1073/pnas.0135855100 12502787 PMC140905

[B118] Meikle P. J. Duplock S. Blacklock D. Whitfield P. D. Macintosh G. Hopwood J. J. (2008). Effect of Lysosomal Storage on Bis(Monoacylglycero)Phosphate. Biochem. J. 411 (1), 71–78. 10.1042/BJ20071043 18052935

[B119] Meneses-Salas E. García-Melero A. Kanerva K. Blanco-Muñoz P. Morales-Paytuvi F. Bonjoch J. (2020). Annexin A6 Modulates TBC1D15/Rab7/StARD3 Axis to Control Endosomal Cholesterol Export in NPC1 Cells. Cell. Mol. Life Sci. 77 (14), 2839–2857. 10.1007/s00018-019-03330-y 31664461 PMC7326902

[B120] Meske V. Erz J. Priesnitz T. Ohm T.-G. (2014). The Autophagic Defect in Niemann-Pick Disease Type C Neurons Differs from Somatic Cells and Reduces Neuronal Viability. Neurobiol. Dis. 64, 88–97. 10.1016/j.nbd.2013.12.018 24412309

[B121] Miquilena-Colina M. E. Lima-Cabello E. Sanchez-Campos S. Garcia-Mediavilla M. V. Fernandez-Bermejo M. Lozano-Rodriguez T. (2011). Hepatic Fatty Acid Translocase CD36 Upregulation Is Associated with Insulin Resistance, Hyperinsulinaemia and Increased Steatosis in Non-alcoholic Steatohepatitis and Chronic Hepatitis C. Gut 60 (10), 1394–1402. 10.1136/gut.2010.222844 21270117

[B122] Mistry P. K. Liu J. Yang M. Nottoli T. McGrath J. Jain D. (2010). Glucocerebrosidase Gene-Deficient Mouse Recapitulates Gaucher Disease Displaying Cellular and Molecular Dysregulation beyond the Macrophage. Proc. Natl. Acad. Sci. 107 (45), 19473–19478. 10.1073/pnas.1003308107 20962279 PMC2984187

[B123] Mitchison N. A. (2004). T-cell-B-cell Cooperation. Nat. Rev. Immunol. 4 (4), 308–312. 10.1038/nri1334 15057789

[B124] Mizunoe Y. Kobayashi M. Hoshino S. Tagawa R. Itagawa R. Hoshino A. (2020). Cathepsin B Overexpression Induces Degradation of Perilipin 1 to Cause Lipid Metabolism Dysfunction in Adipocytes. Sci. Rep. 10 (1), 1–12. 10.1038/s41598-020-57428-6 31959889 PMC6971249

[B125] Mizunoe Y. Kobayashi M. Tagawa R. Nakagawa Y. Shimano H. Higami Y. (2019). Association between Lysosomal Dysfunction and Obesity-Related Pathology: A Key Knowledge to Prevent Metabolic Syndrome. Ijms 20 (15), 3688. 10.3390/ijms20153688 PMC6696452 31357643

[B126] Mizunoe Y. Sudo Y. Okita N. Hiraoka H. Mikami K. Narahara T. (2017). Involvement of Lysosomal Dysfunction in Autophagosome Accumulation and Early Pathologies in Adipose Tissue of Obese Mice. Autophagy 13 (4), 642–653. 10.1080/15548627.2016.1274850 28121218 PMC5388215

[B127] Moreau D. Vacca F. Vossio S. Scott C. Colaco A. Paz Montoya J. (2019). Drug‐induced Increase in Lysobisphosphatidic Acid Reduces the Cholesterol Overload in Niemann-Pick Type C Cells and Mice. EMBO Rep. 20 (7), 1–15. 10.15252/embr.201847055 PMC6607015 31267706

[B128] Nagral A. (2014). Gaucher Disease. J. Clin. Exp. Hepatol. 4 (1), 37–50. 10.1016/j.jceh.2014.02.005 25755533 PMC4017182

[B129] Nakagawa T. Y. Brissette W. H. Lira P. D. Griffiths R. J. Petrushova N. Stock J. (1999). Impaired Invariant Chain Degradation and Antigen Presentation and Diminished Collagen-Induced Arthritis in Cathepsin S Null Mice. Immunity 10 (2), 207–217. 10.1016/s1074-7613(00)80021-7 10072073

[B130] Nascimbeni F. Dalla Salda A. Carubbi F. (2018). Energy Balance, Glucose and Lipid Metabolism, Cardiovascular Risk and Liver Disease Burden in Adult Patients with Type 1 Gaucher Disease. Blood Cel Mol. Dis. 68, 74–80. 10.1016/j.bcmd.2016.10.012 27839982

[B131] Nascimbeni F. Lugari S. Cassinerio E. Motta I. Cavicchioli A. Dalla Salda A. (2020). Liver Steatosis Is Highly Prevalent and Is Associated with Metabolic Risk Factors and Liver Fibrosis in Adult Patients with Type 1 Gaucher Disease. Liver Int. 40 (12), 3061–3070. 10.1111/liv.14640 32810900

[B132] Neuschwander-Tetri B. A. Brent A. (2005). Nonalcoholic Steatohepatitis and the Metabolic Syndrome. Am. J. Med. Sci. 330 (6), 326–335. 10.1097/00000441-200512000-00011 16355018

[B133] Neßlauer A.-M. Gläser A. Gräler M. Engelmann R. Müller-Hilke B. Frank M. (2019). A Therapy With Miglustat, 2-Hydroxypropyl-SS-Cyclodextrin and Allopregnanolone Restores Splenic Cholesterol Homeostasis in Niemann-Pick Disease Type C1. Lipids Health Dis. 18 (1), 146. 10.1186/s12944-019-1088-2 31248418 PMC6598286

[B134] Nguyen Y. Stirnemann J. Lautredoux F. Cador B. Bengherbia M. Yousfi K. (2020). Immunoglobulin Abnormalities in Gaucher Disease: An Analysis of 278 Patients Included in the French Gaucher Disease Registry. Ijms 21 (4), 1247. 10.3390/ijms21041247 PMC7072938 32069933

[B135] Obino D. Diaz J. Sáez J. J. Ibañez-Vega J. Sáez P. J. Alamo M. (2017). Vamp-7-dependent Secretion at the Immune Synapse Regulates Antigen Extraction and Presentation in B-Lymphocytes. MBoC 28 (7), 890–897. 10.1091/mbc.E16-10-0722 28179460 PMC5385938

[B136] Osellame L. D. Rahim A. A. Hargreaves I. P. Richard-Londt A. Brandner S. Waddington S. N. (2013). Mitochondria and Quality Control Defects in a Mouse Model of Gaucher Disease-Links to Parkinson's Disease. Cel Metab. 17 (6), 941–953. 10.1016/j.cmet.2013.04.014 PMC3678026 23707074

[B137] Oyarzún J. E. Lagos J. Vázquez M. C. Valls C. De la Fuente C. Yuseff M. I. (2019). Lysosome Motility and Distribution: Relevance in Health and Disease. Biochim. Biophys. Acta (Bba) - Mol. Basis Dis. 1865 (6), 1076–1087. 10.1016/j.bbadis.2019.03.009 30904612

[B138] Pacheco C. D. Kunkel R. Lieberman A. P. (2007). Autophagy in Niemann-Pick C Disease Is Dependent upon Beclin-1 and Responsive to Lipid Trafficking Defects. Hum. Mol. Genet. 16 (12), 1495–1503. 10.1093/hmg/ddm100 17468177

[B139] Pacheco C. D. Lieberman A. P. (2008). The Pathogenesis of Niemann-Pick Type C Disease: a Role for Autophagy? Expert Rev. Mol. Med. 10 (1), e26. 10.1017/S146239940800080X 18782459 PMC2662713

[B140] Pallottini V. Pfrieger F. W. (2020). Understanding and Treating Niemann-Pick Type C Disease: Models Matter. Ijms 21 (23), 8979. 10.3390/ijms21238979 PMC7730076 33256121

[B141] Pandey M. K. Grabowski G. A. (2013). Immunological Cells and Functions in Gaucher Disease. Crit. Rev. Oncog 18 (3), 197–220. 10.1615/critrevoncog.2013004503 23510064 PMC3661296

[B142] Park H.-J. KimKim D.-H. Kim W.-J. Kim J. Y. Senejani A. G. Hwang S. S. (2014). PPARγ Negatively Regulates T Cell Activation to Prevent Follicular Helper T Cells and Germinal Center Formation. PLoS ONE 9 (6), e99127. 10.1371/journal.pone.0099127 24921943 PMC4055678

[B143] Pascua-Maestro R. Diez-Hermano S. Lillo C. Ganfornina M. D. Sanchez D. (2017). Protecting Cells by Protecting Their Vulnerable Lysosomes: Identification of a New Mechanism for Preserving Lysosomal Functional Integrity upon Oxidative Stress. Plos Genet. 13 (2), e1006603. 10.1371/journal.pgen.1006603 28182653 PMC5325589

[B144] Pejnovic N. N. Pantic J. M. Pantic I. P. I. G. Zdravkovic N. S. Djukic A. L. Arsenijevic N. N. (2013). Galectin-3 Deficiency Accelerates High-Fat Diet-Induced Obesity and Amplifies Inflammation in Adipose Tissue and Pancreatic Islets. Diabetes 62 (6), 1932–1944. 10.2337/db12-0222 23349493 PMC3661611

[B145] Pepino M. Y. Kuda O. Samovski D. Abumrad N. A. (2014). Structure-Function of CD36 and Importance of Fatty Acid Signal Transduction in Fat Metabolism. Annu. Rev. Nutr. 34, 281–303. 10.1146/annurev-nutr-071812-161220 24850384 PMC4329921

[B146] Pergande M. R. Serna‐Perez F. Mohsin S. B. Hanek J. Cologna S. M. (2019). Lipidomic Analysis Reveals Altered Fatty Acid Metabolism in the Liver of the Symptomatic Niemann-Pick, Type C1 Mouse ModelLipidomic Analysis Reveals Altered Fatty Acid Metabolism in the Liver of the Symptomatic Niemann–Pick, Type C1 Mouse Model. PROTEOMICS 19 (18), 1800285. 10.1002/pmic.201800285 31394590

[B147] Pettinelli P. Videla L. A. (2011). Up-Regulation of PPAR-γ mRNA Expression in the Liver of Obese Patients: an Additional Reinforcing Lipogenic Mechanism to SREBP-1c Induction. J. Clin. Endocrinol. Metab. 96 (5), 1424–1430. 10.1016/j.cmet.2008.03.003 21325464

[B148] Peverill W. Powell L. Skoien R. (2014). Evolving Concepts in the Pathogenesis of NASH: Beyond Steatosis and Inflammation. Ijms 15 (5), 8591–8638. 10.3390/ijms15058591 24830559 PMC4057750

[B149] Phipps-Yonas H. Semik V. Hastings K. T. (2013). GILT Expression in B Cells Diminishes Cathepsin S Steady-State Protein Expression and Activity. Eur. J. Immunol. 43 (1), 65–74. 10.1002/eji.201242379 23012103 PMC3706190

[B150] Platt F. M. d’Azzo A. Davidson B. L. Neufeld E. F. Tifft C. J. (2018). Alessandra D’Azzo, Beverly L. Davidson, Elizabeth F. Neufeld, and Cynthia J. TifftLysosomal Storage Diseases. Nat. Rev. Dis. Primers 4 (1), 27. 10.1038/s41572-018-0025-4 30275469

[B151] Platt N. Speak A. O. Colaco A. Gray J. Smith D. A. Williams I. M. (2016). Immune Dysfunction in Niemann-Pick Disease Type C. J. Neurochem. 136, 74–80. 10.1111/jnc.13138 25946402 PMC4833189

[B152] Popkin B. M. Doak C. M. (2009). The Obesity Epidemic Is a Worldwide Phenomenon. Nutr. Rev. 56 (4), 106–114. 10.1111/j.1753-4887.1998.tb01722.x 9584495

[B153] Pribasnig M. A. Mrak I. Grabner G. F. Taschler U. Knittelfelder O. Scherz B. (2015). α/β Hydrolase Domain-Containing 6 (ABHD6) Degrades the Late Endosomal/Lysosomal Lipid Bis(monoacylglycero)phosphate. J. Biol. Chem. 290 (50), 29869–29881. 10.1074/jbc.M115.669168 26491015 PMC4705992

[B154] Prinz W. A. Toulmay A. Balla T. (2020). The Functional Universe of Membrane Contact Sites. Nat. Rev. Mol. Cel Biol. 21 (1), 7–24. 10.1038/s41580-019-0180-9 PMC10619483 31732717

[B155] Rada P. González-Rodríguez Á. García-Monzón C. Valverde Á. M. Valverde Á. M (2020). Understanding Lipotoxicity in NAFLD Pathogenesis: Is CD36 a Key Driver? Cel Death Dis. 11 (9), 802. 10.1038/s41419-020-03003-w PMC7519685 32978374

[B156] Ramon S. Bancos S. Thatcher T. H. Murant T. I. Moshkani S. Sahler J. M. (2012). Peroxisome Proliferator-Activated Receptor γ B Cell-Specific-Deficient Mice Have an Impaired Antibody Response. J.I. 189 (10), 4740–4747. 10.4049/jimmunol.1200956 PMC3490033 23041568

[B157] Rawnsley D. R. Diwan A. (2020). Lysosome Impairment as a Trigger for Inflammation in Obesity: The Proof Is in the Fat. EBioMedicine 56, 102824. 10.1016/j.ebiom.2020.102824 32540774 PMC7300142

[B158] Rieusset J. (2017). Endoplasmic Reticulum-Mitochondria Calcium Signaling in Hepatic Metabolic Diseases. Biochim. Biophys. Acta (Bba) - Mol. Cel Res. 1864 (6), 865–876. 10.1016/j.bbamcr.2017.01.001 28064001

[B159] Rigante D. Cipolla C. Basile U. Gulli F. Savastano M. C. (2017). Overview of Immune Abnormalities in Lysosomal Storage Disorders. Immunol. Lett. 188 (July), 79–85. 10.1016/j.imlet.2017.07.004 28687233

[B160] Roche P. A. Cresswell P. (1990). Invariant Chain Association with HLA-DR Molecules Inhibits Immunogenic Peptide Binding. Nature 345 (6276), 615–618. 10.1038/345615a0 2190094

[B161] Russo L. Lumeng C. N. (2018). Properties and Functions of Adipose Tissue Macrophages in Obesity. Immunology 155 (4), 407–417. 10.1111/imm.13002 30229891 PMC6230999

[B162] Sáez J. J. Diaz J. Ibañez J. Bozo J. P. Cabrera Reyes F. Alamo M. (2019). The Exocyst Controls Lysosome Secretion and Antigen Extraction at the Immune Synapse of B Cells. J. Cel Biol. 218 (7), 2247–2264. 10.1083/jcb.201811131 PMC6605794 31197029

[B163] Saito T. Kuma A. Sugiura Y. Ichimura Y. Obata M. Kitamura H. (2019). Autophagy Regulates Lipid Metabolism through Selective Turnover of NCoR1. Nat. Commun. 10 (1). 10.1038/s41467-019-08829-3 PMC6450892 30952864

[B164] Sarkar S. Carroll B. Buganim Y. Maetzel D. Ng A. H. M. Cassady J. P. (2013). Impaired Autophagy in the Lipid-Storage Disorder Niemann-Pick Type C1 Disease. Cel Rep. 5 (5), 1302–1315. 10.1016/j.celrep.2013.10.042 PMC3957429 24290752

[B165] Seppala-Lindroos A. (2002). Fat Accumulation in the Liver Is Associated with Defects in Insulin Suppression of Glucose Production and Serum Free Fatty Acids Independent of Obesity in Normal Men. J. Clin. Endocrinol. Metab. 87 (7), 3023–3028. 10.1210/jcem.87.7.8638 12107194

[B166] Seranova E. Connolly K. J. Zatyka M. Rosenstock T. R. Barrett T. Tuxworth R. I. (2017). Dysregulation of Autophagy as a Common Mechanism in Lysosomal Storage Diseases. Essays Biochem. 61 (6), 733–749. 10.1042/EBC20170055 29233882 PMC5869865

[B167] Serrano-Puebla A. Boya P. (2016). Lysosomal Membrane Permeabilization in Cell Death: New Evidence and Implications for Health and Disease. Ann. N.Y. Acad. Sci. 1371 (1), 30–44. 10.1111/nyas.12966 26599521

[B168] Shaikh S. R. Haas K. M. Beck M. A. Teague H. (2015). The Effects of Diet-Induced Obesity on B Cell Function. Clin. Exp. Immunol. 179 (1), 90–99. 10.1111/cei.12444 25169121 PMC4260901

[B169] Shi L. Liu J. Su Qing. Yang Zhen. (2019). Glucose Metabolism Imaging. Front. Endocrinol. 10 (October), 1–10. 10.1007/978-3-319-48382-5_810.1007/978-981-13-7458-6_1

[B170] Showalter M. R. Berg A. L. Nagourney A. Heil H. Carraway K. L. Fiehn O. (2020). The Emerging and Diverse Roles of Bis(Monoacylglycero) Phosphate Lipids in Cellular Physiology and Disease. Ijms 21 (21), 8067. 10.3390/ijms21218067 PMC7663174 33137979

[B171] Silverstein R. L. Febbraio. M. (2009). CD36, a Scavenger Receptor Involved in Immunity, Metabolism, Angiogenesis, and Behavior. Sci. Signal. 2 (72). 10.1126/scisignal.272re3 PMC2811062 19471024

[B172] Singh R. Cresswell P. (2010). Defective Cross-Presentation of Viral Antigens in GILT-free Mice. Science 328 (5984), 1394–1398. 10.1126/science.1189176 20538950 PMC2925227

[B173] Singh R. Xiang Y. Wang Y. Baikati K. Cuervo A. M. Luu Y. K. (2009). Autophagy Regulates Adipose Mass and Differentiation in Mice. J. Clin. Invest. 119 (11), 3329–3339. 10.1172/JCI39228 19855132 PMC2769174

[B174] Singhal A. Krystofiak E. S. Jerome W. G. Song B. (2020). 2-Hydroxypropyl-Gamma-Cyclodextrin Overcomes NPC1 Deficiency by Enhancing Lysosome-ER Association and Autophagy. Sci. Rep. 10 (1), 1–14. 10.1038/s41598-020-65627-4 32457374 PMC7250861

[B175] Singhal A. Szente L. Hildreth J. E. K. Song B. (2018). Hydroxypropyl-beta and -gamma Cyclodextrins rescue Cholesterol Accumulation in Niemann-Pick C1 Mutant Cell via Lysosome-Associated Membrane Protein 1. Cel Death Dis. 9 (10), 1019. 10.1038/s41419-018-1056-1 PMC6170477 30282967

[B176] Soccio R. E. Breslow J. L. (2004). Intracellular Cholesterol Transport. Atvb 24 (7), 1150–1160. 10.1161/01.ATV.0000131264.66417.d5 15130918

[B177] Soussi H. Clément K. Dugail I. (2016). Adipose Tissue Autophagy Status in Obesity: Expression and Flux-Two Faces of the Picture. Autophagy 12 (3), 588–589. 10.1080/15548627.2015.1106667 26565777 PMC4835957

[B178] Srikakulapu P. McNamara C. A. (2020). B Lymphocytes and Adipose Tissue Inflammation. Atvb 40 (5), 1110–1122. 10.1161/ATVBAHA.119.312467 PMC7398177 32131612

[B179] Starosta R. T. Vairo F. P. e. Dornelles A. D. Basgalupp S. P. Siebert M. Pedroso M. L. A. (2020). Liver Involvement in Patients with Gaucher Disease Types I and III. Mol. Genet. Metab. Rep. 22 (September 2019), 100564. 10.1016/j.ymgmr.2019.100564 32099816 PMC7026612

[B180] Stirnemann J. Belmatoug N. Camou F. Serratrice C. Froissart R. Caillaud C. (2017). A Review of Gaucher Disease Pathophysiology, Clinical Presentation and Treatments. Ijms 18 (2), 441. 10.3390/ijms18020441 PMC5343975 28218669

[B181] Sun Y. Liou B. Ran H. Skelton M. R. Williams M. T. Vorhees C. V. (2010). Neuronopathic Gaucher Disease in the Mouse: Viable Combined Selective Saposin C Deficiency and Mutant Glucocerebrosidase (V394L) Mice with Glucosylsphingosine and Glucosylceramide Accumulation and Progressive Neurological Deficits. Hum. Mol. Genet. 19 (6), 1088–1097. 10.1093/hmg/ddp580 20047948 PMC2830832

[B182] Tatti M. Motta M. Di bartolomeo S. Scarpa S. Cianfanelli V. Cecconi F. (2012). Reduced Cathepsins B and D Cause Impaired Autophagic Degradation that Can Be Almost Completely Restored by Overexpression of These Two Proteases in Sap C-Deficient Fibroblasts. Hum. Mol. Genet. 21 (23), 5159–5173. 10.1093/hmg/dds367 22949512

[B183] te Vruchte D. Jeans A. Platt F. M. Sillence D. J. (2010). Glycosphingolipid Storage Leads to the Enhanced Degradation of the B Cell Receptor in Sandhoff Disease Mice. J. Inherit. Metab. Dis. 33 (3), 261–270. 10.1007/s10545-010-9109-3 20458542 PMC3779831

[B184] Tian K. Xu Y. Sahebkar A. Xu S. (2020). CD36 in Atherosclerosis: Pathophysiological Mechanisms and Therapeutic Implications. Curr. Atheroscler. Rep. 22 (10). 10.1007/s11883-020-00870-8 32772254

[B185] Tong L. Wang L. Yao S. Jin L. Yang J. Zhang Y. (2019). PPARδ Attenuates Hepatic Steatosis through Autophagy-Mediated Fatty Acid Oxidation. Cel Death Dis. 10 (3). 10.1038/s41419-019-1458-8 PMC6393554 30814493

[B186] Tontonoz P. Spiegelman B. M. (2008). Fat and beyond: The Diverse Biology of PPARγ. Annu. Rev. Biochem. 77, 289–312. 10.1146/annurev.biochem.77.061307.091829 18518822

[B187] Turcot V. Bouchard L. Faucher G. Tchernof A. Deshaies Y. Pérusse L. (2012). A Polymorphism of the Interferon-Gamma-Inducible Protein 30 Gene Is Associated with Hyperglycemia in Severely Obese Individuals. Hum. Genet. 131 (1), 57–66. 10.1007/s00439-011-1043-4 21701784

[B188] Turk V. (2001). New Embo Members' Review: Lysosomal Cysteine Proteases: Facts and Opportunities. EMBO J. 20 (17), 4629–4633. 10.1093/emboj/20.17.4629 11532926 PMC125585

[B189] Turk V. Stoka V. Vasiljeva O. Renko M. Sun T. Turk B. (2012). Cysteine Cathepsins: From Structure, Function and Regulation to New Frontiers. Biochim. Biophys. Acta (Bba) - Proteins Proteomics 1824 (1), 68–88. 10.1016/j.bbapap.2011.10.002 22024571 PMC7105208

[B190] Uranga R. M. Keller J. N. (2019). The Complex Interactions between Obesity, Metabolism and the Brain. Front. Neurosci. 13, 1–21. 10.3389/fnins.2019.00513 31178685 PMC6542999

[B191] Urban B. C. Willcox N. Roberts D. J. (2001). A Role for CD36 in the Regulation of Dendritic Cell Function. Proc. Natl. Acad. Sci. 98 (15), 8750–8755. 10.1073/pnas.151028698 11447263 PMC37507

[B192] van der Lienden M. J. C. Aten J. Marques A. R. A. Marques I. S. E. Larsen P. W. B. Claessen N. (2021). Gcase and Limp2 Abnormalities in the Liver of Niemann Pick Type C Mice. Ijms 22 (5), 2532. 10.3390/ijms22052532 33802460 PMC7959463

[B193] Vanier M. Millat G. (2003). Niemann-Pick Disease Type C. Clin. Genet. 64 (4), 269–281. 10.1034/j.1399-0004.2003.00147.x 12974729

[B194] Vázquez M. C. del Pozo T. Robledo F. A. Carrasco G. Pavez L. Olivares F. (2011). Talía del Pozo, Fermín A. Robledo, Gonzalo Carrasco, Leonardo Pavez, Felipe Olivares, Mauricio González, and Silvana ZanlungoAlteration of Gene Expression Profile in Niemann-Pick Type C Mice Correlates with Tissue Damage and Oxidative Stress. PLoS ONE 6 (12), e28777. 10.1371/journal.pone.0028777 22216111 PMC3245218

[B195] Vázquez-Vela M. E. F. Torres N. Tovar A. R. Tovar A. R. (2008). White Adipose Tissue as Endocrine Organ and its Role in Obesity. Arch. Med. Res. 39 (8), 715–728. 10.1016/j.arcmed.2008.09.005 18996284

[B196] Vidal-Puig A. J. Considine R. V. Jimenez-Liñan M. Werman A. Pories W. J. Caro J. F. (1997). Peroxisome Proliferator-Activated Receptor Gene Expression in Human Tissues. Effects of Obesity, Weight Loss, and Regulation by Insulin and Glucocorticoids. J. Clin. Invest. 99 (10), 2416–2422. 10.1172/JCI119424 9153284 PMC508081

[B197] Vitner E. B. Dekel H. Zigdon H. Shachar T. Farfel-Becker T. Eilam R. (2010). Hani Dekel, Hila Zigdon, Tamar Shachar, Tamar Farfel-Becker, Raya Eilam, Stefan Karlsson, and Anthony H. FutermanAltered Expression and Distribution of Cathepsins in Neuronopathic Forms of Gaucher Disease and in Other Sphingolipidoses. Hum. Mol. Genet. 19 (18), 3583–3590. 10.1093/hmg/ddq273 20616152

[B198] Wang F. Jia J. Rodrigues B. (2017). Autophagy, Metabolic Disease, and Pathogenesis of Heart Dysfunction. Can. J. Cardiol. 33 (7), 850–859. 10.1016/j.cjca.2017.01.002 28389131

[B199] Wang T. Wei Q. Liang L. Tang X. Yao J. Lu Y. (2020a). OSBPL2 Is Required for the Binding of COPB1 to ATGL and the Regulation of Lipid Droplet Lipolysis. IScience 23 (7), 101252. 10.1016/j.isci.2020.101252 32650117 PMC7348002

[B200] Wang Y. Nakajima T. Gonzalez F. J. Tanaka N. (2020b). PPARs as Metabolic Regulators in the Liver: Lessons from Liver-specific PPAR-Null Mice. Ijms 21 (6), 2061. 10.3390/ijms21062061 PMC7139552 32192216

[B201] Weisberg S. P. McCann D. Desai M. Rosenbaum M. Leibel R. L. Ferrante A. W. (2003). Daniel McCann, Manisha Desai, Michael Rosenbaum, Rudolph L. Leibel, and Anthony W. FerranteObesity Is Associated with Macrophage Accumulation in Adipose Tissue. J. Clin. Invest. 112 (12), 1796–1808. 10.1172/JCI1924610.1172/jci200319246 14679176 PMC296995

[B202] Winer D. A. Winer S. Shen L. Wadia P. P. Yantha J. Paltser G. (2011). B Cells Promote Insulin Resistance through Modulation of T Cells and Production of Pathogenic IgG Antibodies. Nat. Med. 17 (5), 610–617. 10.1038/nm.2353 21499269 PMC3270885

[B203] Won W.-J. Bachmann M. F. Kearney J. F. (2008). CD36 Is Differentially Expressed on B Cell Subsets during Development and in Responses to Antigen. J. Immunol. 180 (1), 230–237. 10.4049/jimmunol.180.1.230 18097024

[B204] Wraith J. E. Baumgartner M. R. Bembi B. Covanis A. Levade T. Mengel E. (2009). Recommendations on the Diagnosis and Management of Niemann-Pick Disease Type C. Mol. Genet. Metab. 98 (1–2), 152–165. 10.1016/j.ymgme.2009.06.008 19647672

[B205] Wu Z. Xu J. Tan J. Song Y. Liu L. Zhang F. (2019). Mesenteric Adipose Tissue B Lymphocytes Promote Local and Hepatic Inflammation in Non‐alcoholic Fatty Liver Disease Mice. J. Cel Mol. Med. 23 (5), 3375–3385. 10.1111/jcmm.14232 PMC6484337 30772951

[B206] Xie C. Turley S. D. Dietschy J. M. (2000). Centripetal Cholesterol Flow from the Extrahepatic Organs through the Liver Is Normal in Mice with Mutated Niemann-Pick Type C Protein (NPC1). J. Lipid Res. 41 (8), 1278–1289. PMID: 10946016. 10.1016/s0022-2275(20)33436-2 10946016

[B207] Xu Q. Mariman E. C. M. Goossens G. H. Blaak E. E. Jocken J. W. E. JohanJocken W. E. (2020). Cathepsin Gene Expression in Abdominal Subcutaneous Adipose Tissue of Obese/Overweight Humans. Adipocyte 9 (1), 246–252. 10.1080/21623945.2020.1775035 32486882 PMC7469552

[B208] Yadati T. Houben T. Bitorina A. Oligschlaeger Y. Gijbels M. J. Mohren R. (2021). Inhibition of Extracellular Cathepsin D Reduces Hepatic Lipid Accumulation and Leads to Mild Changes in Inflammationin NASH Mice. Front. Immunol. 12 (July), 675535. 10.3389/fimmu.2021.675535 34335574 PMC8323051

[B209] Yadati T. Houben T. Bitorina A. Shiri-Sverdlov R. (2020). The Ins and Outs of Cathepsins: Physiological Function and Role in Disease Management. Cells 9 (7), 1679. 10.3390/cells9071679 PMC7407943 32668602

[B210] Yañez M. J. Marín T. Balboa E. Klein A. D. Alvarez A. R. Zanlungo S. (2020). Finding Pathogenic Commonalities between Niemann-Pick Type C and Other Lysosomal Storage Disorders: Opportunities for Shared Therapeutic Interventions. Biochim. Biophys. Acta (Bba) - Mol. Basis Dis. 1866 (10), 165875. 10.1016/j.bbadis.2020.165875 32522631

[B211] Yang L. Li P. Fu S. Calay E. S. Hotamisligil G. S. (2010). Defective Hepatic Autophagy in Obesity Promotes ER Stress and Causes Insulin Resistance. Cel Metab. 11 (6), 467–478. 10.1016/j.cmet.2010.04.005 PMC2881480 20519119

[B212] Yazıcı D. Sezer H. (2017). “Insulin Resistance, Obesity and Lipotoxicity,” in ” in Vol. 960. Advances in Experimental Medicine and Biology. Editors Engin A. B. Engin A. (Cham: Springer International Publishing), 277–304. 10.1007/978-3-319-48382-5_12

[B213] You Y. Bao W.-L. Zhang S.-L. Li H.-D. Li H. Dang W.-Z. (2020). Sorting Nexin 10 Mediates Metabolic Reprogramming of Macrophages in Atherosclerosis through the Lyn-dependent TFEB Signaling Pathway. Circ. Res. 127 (4), 534–549. 10.1161/CIRCRESAHA.119.315516 32316875

[B214] Yu H. Yang F. Zhong W. Jiang X. Zhang F. Ji X. (2021). Secretory Galectin-3 Promotes Hepatic Steatosis via Regulation of the PPARγ/CD36 Signaling Pathway. Cell Signal. 84 (May), 110043. 10.1016/j.cellsig.2021.110043 33991615

[B215] Yu W. Gong J.-S. Ko M. Garver W. S. Yanagisawa K. Michikawa M. (2005). Altered Cholesterol Metabolism in Niemann-Pick Type C1 Mouse Brains Affects Mitochondrial Function. J. Biol. Chem. 280 (12), 11731–11739. 10.1074/jbc.M412898200 15644330

[B216] Yuseff M.-I. Lennon-DumÃ©nil A. M. Ana M. L. (2015). B Cells Use Conserved Polarity Cues to Regulate Their Antigen Processing and Presentation Functions. Front. Immunol. 6 (4), 1–7. 10.3389/fimmu.2015.00251 26074919 PMC4445385

[B217] Yuseff M.-I. Reversat A. Lankar D. Diaz J. Fanget I. Pierobon P. (2011). Polarized Secretion of Lysosomes at the B Cell Synapse Couples Antigen Extraction to Processing and Presentation. Immunity 35 (3), 361–374. 10.1016/j.immuni.2011.07.008 21820334

[B218] Zhang F. Jiang W. W. Li X. Qiu X. Y. Wu Z. Chi Y. J. (2016). Role of Intrahepatic B Cells in Non-alcoholic Fatty Liver Disease by Secreting Pro-inflammatory Cytokines and Regulating Intrahepatic T Cells. J. Dig. Dis. 17 (7), 464–474. 10.1111/1751-2980.12362 27216040

[B219] Zhang X. Evans T. D. Jeong S.-J. Razani B. (2018b). Classical and Alternative Roles for Autophagy in Lipid Metabolism. Curr. Opin. Lipidol. 29 (3), 203–211. 10.1097/MOL.0000000000000509 29601311 PMC5930069

[B220] Zhang Y. Sowers J. R. Ren. J. (2018a). Targeting Autophagy in Obesity: From Pathophysiology to Management. Nat. Rev. Endocrinol. 14 (6), 356–376. 10.1038/s41574-018-0009-1 29686432

[B221] Zhao L. Zhang C. Luo X. Wang P. Zhou W. Zhong S. (2018). CD36 Palmitoylation Disrupts Free Fatty Acid Metabolism and Promotes Tissue Inflammation in Non-alcoholic Steatohepatitis. J. Hepatol. 69 (3), 705–717. 10.1016/j.jhep.2018.04.006 29705240

